# A novel HSP90 inhibitor SL-145 suppresses metastatic triple-negative breast cancer without triggering the heat shock response

**DOI:** 10.1038/s41388-022-02269-y

**Published:** 2022-05-02

**Authors:** Ji Young Kim, Tae-Min Cho, Jung Min Park, Soeun Park, Minsu Park, Kee Dal Nam, Dongmi Ko, Juyeon Seo, Seongjae Kim, Eunsun Jung, Lee Farrand, Cong-Truong Nguyen, Van-Hai Hoang, Minh Thanh La, Jihyae Ann, Gibeom Nam, Hyun-Ju Park, Jeewoo Lee, Yoon-Jae Kim, Jae Hong Seo

**Affiliations:** 1 Division of Medical Oncology, Department of Internal Medicine, Korea University College of Medicine, Korea University, Seoul, 02841 Republic of Korea; 2 Department of Biomedical Research Center, Korea University Guro Hospital, Korea University, Seoul, 08308 Republic of Korea; 3 Brain Korea 21 Program for Biomedical Science, Korea University College of Medicine, Korea University, Seoul, 02841 Republic of Korea; 4 Adelaide Medical School, Faculty of Health and Medical Sciences, The University of Adelaide, 5000 Adelaide, SA Australia; 5 Laboratory of Medicinal Chemistry, College of Pharmacy, Seoul National University, Seoul, 08826 Republic of Korea; 6 School of Pharmacy, Sungkyunkwan University, Suwon, Gyeonggi-do 16419 Republic of Korea

**Keywords:** Breast cancer, Apoptosis

## Abstract

Despite recent advances, there remains a significant unmet need for the development of new targeted therapies for triple-negative breast cancer (TNBC). Although the heat shock protein HSP90 is a promising target, previous inhibitors have had issues during development including undesirable induction of the heat shock response (HSR) and off-target effects leading to toxicity. SL-145 is a novel, rationally-designed C-terminal HSP90 inhibitor that induces apoptosis in TNBC cells via the suppression of oncogenic AKT, MEK/ERK, and JAK2/STAT3 signaling and does not trigger the HSR, in contrast to other inhibitors. In an orthotopic allograft model incorporating breast cancer stem cell-enriched TNBC tumors, SL-145 potently suppressed tumor growth, angiogenesis, and metastases concomitant with dysregulation of the JAK2/STAT3 signaling pathway. Our findings highlight the potential of SL-145 in suppressing metastatic TNBC independent of the HSR.

## Introduction

Triple-negative breast cancer (TNBC) is the most aggressive and lethal subtype of breast cancer with high heterogeneity, associated with increased risks of recurrence and metastasis [1]. Standard treatment for TNBC patients relies on broadly cytotoxic anticancer drugs due to the absence of commercially-available targeted therapies [2, 3]. Despite numerous clinical trials for single and combination therapies of the PI3K/AKT, MEK/ERK, JAK2/STAT3, and mTOR signaling pathways, none have yet resulted in an improvement in overall survival for TNBC patients within an acceptable therapeutic window [1–4]. Evidence suggests that breast cancer stem cells (BCSCs) harboring tumor-initiating potential and self-renewal capacity may be at least partially responsible for such poor clinical outcomes [3, 5, 6]. Although chemo- and radiation therapies are often successful at destroying a large proportion of the tumor bulk, recurrence is thought to occur due to the presence of these subpopulations and their targeting may be essential to elimination of the entire tumor cell population.

Heat shock protein 90 (HSP90) is a molecular chaperone that maintains the structural and functional integrity of numerous client proteins many of which have oncogenic potential, including AKT/MEK/JAK2/STAT3 and pluripotent transcription factors [7–9]. Over the last two decades, the development of HSP90 inhibitors has largely focused on targeting the N-terminal ATP-binding domain, however, none have succeeded to date [10–12]. In general, HSP90 inhibitors targeting the N-terminus trigger translocation of the transcription factor HSF-1 to the nucleus, leading to further upregulation of HSP70, HSP90, and other HSPs, followed by activation of the survival cascade known as the heat shock response (HSR) [10, 13–15]. HSP70 is known to be a pro-survival factor that hinders the induction of apoptosis, leading to drug resistance [7, 16]. We sought to determine whether targeting the C-terminal domain of HSP90 could lead to effective suppression of oncogenic signaling while avoiding induction of the pro-survival HSR.

## Material and methods

### Materials used (details listed in Supplementary Information)

Reagents, materials and antibodies, cell viability assay, annexin V/PI assay, caspase-3 activity assay, CD44^high^/CD24^low^ staining, aldefluor-positivity assay, immunoblot analysis, HSP90α (C-terminal) inhibitor screening assay, N-terminal HSP90 binding activity assay, chromatin immunoprecipitation (ChIP) assay, real-time quantitative polymerase chain reaction (RT-qPCR) analysis, reverse transcription PCR analysis, immunocytochemistry, molecular modeling, and docking analysis, allograft in vivo experiments and bioluminescence imaging, immunohistochemistry and in-situ localization of apoptosis (TUNEL), mammosphere formation in vitro and in vivo assays, MMP-2 and MMP-9 ELISA assay, wound healing assay and serum biochemistry profiles for biomarkers of liver and renal injury.

### Breast cancer cell culture

The TNBC cell lines MDA-MB-231 (PerkinElmer, Inc. CT), Hs578T (American Type Culture Collection, ATCC), BT549, and 4T1-Luc (Japanese Collection of Research Bioresources Cell Bank, JCRB), the normal murine mammary gland epithelial cell line NMuMG (ATCC) and the normal human embryonic kidney cell line HEK293 (JCRB) were cultured in MEM or RPMI 1640 (Gibco, MD) containing 10% fetal bovine serum (FBS), and streptomycin-penicillin (100 U/ml). Normal human mammary epithelial MCF10A (ATCC) cells were cultured in Mammary Epithelial Cell Growth Medium (MEGM), including hEGF, insulin, hydrocortisone, and bovine pituitary extract (SingleQuotsTM Kit, Lonza, CA) containing streptomycin-penicillin (100 U/ml). Cells were incubated at 37 °C in an atmosphere of 5% CO_2_. All cell lines were authenticated by short tandem repeat (STR) profiling by Macrogen Inc (Seoul, South Korea).

### Allograft in vivo experiments and bioluminescence imaging

All animal procedures were carried out in accordance with animal care guidelines approved by the Korea University Institutional Animal Care and Use Committee (IACUC, KOREA-2018-0135). Five-week-old female BALB/c mice were obtained from the Shizuoka Laboratory Animal Center (Shizuoka, Japan) and housed in a specific pathogen-free environment. The animals were acclimated for 1 week prior to the study and had free access to food and water. 1 × 10^5^ cells from 4T1 mammospheres were implanted subcutaneously in the right flank of 6-week-old BALB/c female mice (*n* = 8/each group). After 1 week, vehicle (DMSO/corn oil, 1:9) or SL-145 (20 mg/kg/day, every other day) was administered intraperitoneally for 27 days, and tumor volumes were measured using a caliper and calculated using the formula V = (Length × Width2)/2. The animals were then anesthetized and subjected to NightOWL LB983 bioluminescence imaging (BLI) (Berthold Technologies, TN). D-luciferin sodium salt (BioVision Inc. Milpitas, CA) at 150 mg/kg body weight in 100 µl PBS was administered intraperitoneally as a substrate before imaging. The captured images were quantified (photons/sec) using the IndiGo™ software package. See “Supplementary Material and Methods” for further details.

### Statistical analysis

All data were analyzed using GraphPad Prism 5.0 statistical software (San Diego, CA). The results are presented as mean ± SEM of at least three independent experiments. Data were analyzed by student’s *t*-test, and one- or two-way ANOVA as appropriate. Significance between multiple experimental groups was determined using the Bonferroni’s post hoc test. The comparison of survival curves was analyzed by log-rank (Mantel-Cox) test. Statistical significance was defined at *p* < 0.05 (*).

## Results and Discussion

### Cytotoxic effect of SL-145 in TNBC cells is associated with suppression of AKT/MEK/ERK activity and caspase-3 activation

**Fig. 1:**
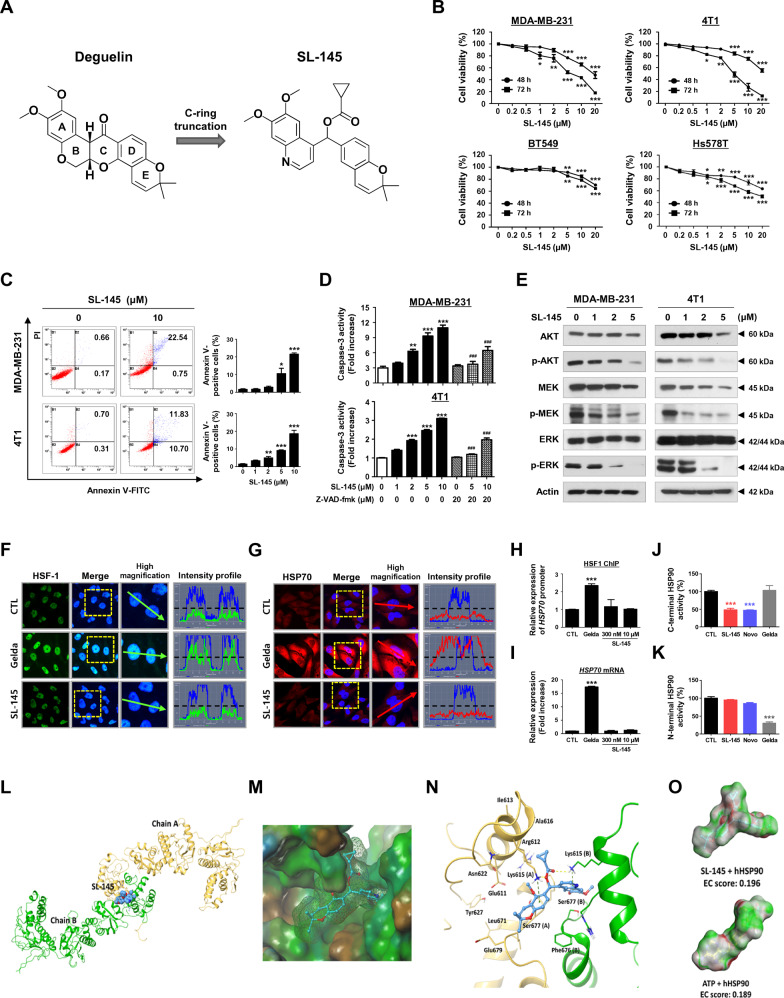
SL-145 effectively kills TNBC cells by targeting the C-terminal binding site of HSP90. **A** Chemical structures of deguelin and SL-145. SL-145 was synthesized as a C-ring truncated deguelin derivative. **B** Effect of SL-145 on cell viability in TNBC cells. MDA-MB-231, BT549, Hs578T, and 4T1 cells were treated at the indicated concentrations of SL-145 (0-20 μM) for 48 and 72 h. Cell viability was determined by MTS assay (*p < 0.05, **p < 0.01, ***p < 0.001, versus DMSO control, *n* = 4). **C** Early and late apoptotic cells in the presence or absence of SL-145 were quantified by annexin V/PI staining (right panel, *p < 0.05, **p < 0.01, ***p < 0.001, *n* = 3). **D** Effect of SL-145 on caspase-3 activity (**p < 0.01, ***p < 0.001, DMSO vs SL-145; ###*p* < 0.001, SL-145 alone vs co-treatment with Z-VAD-fmk, *n* = 3). **E** Immunoblot analyses of AKT, phospho-AKT (Ser473), MEK, phospho-MEK (Ser218/222), ERK, and phospho-ERK (Thr202/Tyr204) protein expression in MDA-MB-231 and 4T1 cells after treatment with SL-145 (0–5 µM, 72 h). **F**–**G** Comparison of the effects of SL-145 or geldanamycin on induction of the heat shock response. MDA-MB-231 cells were immunostained for HSF-1 (green, **F**) or HSP70 (red, **G**) with DAPI (nuclei, blue) following exposure to SL-145 (300 nM) or geldanamycin (300 nM) for 24 h. Fluorescence intensity of these proteins is represented in arbitrary units as defined by the software using the intensity profile tool. **H** Effects of SL-145 and geldanamycin on HSF-1 binding to the HSP70 promoter. MDA-MB-231 cells were treated with SL-145 (300 nM and 10 µM) or geldanamycin (300 nM) for 24 h, and then analyzed for binding of HSF-1 to the HSP70 promoter by ChIP assay. DNA fragments of the HSP70 gene (HSE1, −221 to −114) were amplified by RT-PCR. The relative promoter expression was calculated as fold increase compared to the control group (***p < 0.001, *n* = 4). **I** Relative expression of HSP70 mRNA was analyzed by quantitative RT-PCR in MDA-MB-231 cells following exposure to SL-145 (300 nM and 10 µM) or geldanamycin (300 nM) for 24 h (***p < 0.001, *n* = 4). **J** Effect of SL-145 on inhibition of the C-terminal domain of HSP90. The inhibitory effect of HSP90 inhibitors (SL-145, novobiocin, or geldanamycin, 500 μM) on HSP90α (C-terminal): PPID binding activity was determined by an HSP90α (C-terminal) inhibitor screening assay (***p < 0.001, *n* = 3). Gelda, geldanamycin; Novo, novobiocin; PPID, peptidylprolyl isomerase D. **K** Influence of SL-145 on the N-terminal HSP90 binding activity. The competitive HSP90α binding activity of HSP90 inhibitors (SL-145, novobiocin or geldanamycin, 1000 nM) with FITC-labeled geldanamycin was determined with an HSP90α N-terminal domain assay (***p < 0.001, *n* = 3). The results are presented as mean ± SEM of at least three independent experiments analyzed by Student's *t*-test, one- or two-way ANOVA followed by Bonferroni’s post hoc test. **L–O** Structural modeling of docking between SL-145 and hHSP90. **L** Binding pose of SL-145 in the dimerization interface of open state hHSP90 (Surflex-Dock score (-log Kd) = 7.238). Chain A of hHSP90 is rendered as a yellow ribbon, and chain B is the green ribbon. SL-145 is depicted as a space-filling model. **M** Lipophilicity property surface map (brown: hydrophobic, blue: hydrophilic) of the active site. The Connolly surface of SL-145 is shown as green mesh. **N** Intermolecular interactions between SL-145 and hHSP90. Key amino acid residues within the binding pocket are rendered as capped sticks. Hydrogen bonding (<2.8 Å) and cation-π interactions are represented as yellow and green dashed lines, respectively. **O** Electrostatic complementarity (EC) surface and score for ATP and SL-145. Green = perfect electrostatic complementarity (1), white = both potentials zero, red = perfect electrostatic clash (−1).

We have newly synthesized a novel C-terminal HSP90 inhibitor, SL-145, comprising a C-ring truncated deguelin derivative (Fig. 1A, Supplementary information; synthesis and purity of SL-145). We examined the effect of SL-145 on cell viability in three human TNBC cell lines, MDA-MB-231, BT549, and Hs578T [17] as well as one murine carcinoma cell line (4T1) which spontaneously produces highly metastatic tumors [18]. A concentration-dependent impairment of cell viability was observed in TNBC cells in the presence of SL-145 (Fig. 1B). SL-145 induces apoptosis in MDA-MB-231 and 4T1 cells (Fig. 1C and Supplementary Fig. S1), associated with caspase-3 activation (Fig. 1D). SL-145 (5 µM, 72 h) did not induce apoptosis in normal human mammary epithelial MCF10A, murine mammary gland NMuMG or human embryonic kidney HEK293 cells (Supplementary Fig. S2). Furthermore, we examined cytotoxic effects of the C-terminal HSP90 inhibitors SL-145 and novobiocin, and the N-terminal HSP90 inhibitor geldanamycin in MCF10A cells in vitro. Geldanamycin elicited a significant inhibitory effect on cell viability in MCF10A cells at low concentration (0.01 µM), while both SL-145 and novobiocin had no significant cytotoxicity at 10 µM, a 1000-fold higher concentration (Supplementary Fig. S3).

HSP90 client oncoproteins AKT, MEK and ERK are pro-survival factors that can be aberrantly activated in TNBC, promoting tumor growth and drug resistance [1, 5, 16]. In addition, treatment with dual inhibitors of PI3K/AKT and MAPK has been shown to induce cell cycle arrest and apoptosis in TNBC cells, resulting in a synergistic inhibition of cell growth [19]. Exposure to SL-145 markedly downregulated AKT, MEK, and ERK expression and reduced their phosphorylation (Fig. 1E and Supplementary Fig. S4), suggesting the simultaneous depletion of multiple cellular signaling pathways could contribute to enhanced cytotoxicity against TNBC.

### SL-145 targets the C-terminal domain of HSP90 without triggering the HSR

Oncogenic heat shock factor 1 (HSF-1) is a major transcriptional regulator and has been implicated in multiple aspects of cancer progression including carcinogenesis, drug resistance, and tumor propagation. Activation of the HSF-1/HSP70 axis is a major pathway for HSR induction [14, 20, 21]. C-terminal HSP90 blockade by SL-145 did not induce nuclear accumulation of HSF-1 nor HSP70 upregulation in MDA-MB-231 (Fig. 1F, G, Supplementary Fig. S5, and S6), suggesting that SL-145 impedes HSP90 activity without triggering HSF-1 transcriptional activity. Furthermore, we sought to confirm whether HSF-1 binds to the HSP70 promoter in the presence of SL-145 or geldanamycin using a ChIP assay. No increase in relative expression of the HSP70 promoter (HSE1, −221 to −114) was observed following exposure to SL-145, whereas geldanamycin significantly increased expression of the HSP70 promoter bound to HSF-1 (Fig. 1H and Supplementary Fig. S7). The expression of HSP70 mRNA was subsequently upregulated by geldanamycin treatment (Fig. 1I).

HSP90 consists of an N-terminal ATP-binding domain, the middle domain containing the binding site for the client proteins and co-chaperones, and a C-terminal domain required for dimerization [22]. The C-terminus of HSP90 also contains a highly conserved five amino acid sequence (MEEVD) that selectively interacts with several co-chaperones, including peptidyl-prolyl cis-trans isomerases (PPIase) such as FK506-binding protein 51 (FKBP51), FKBP52, and peptidylprolyl isomerase D (PPID, also known as Cyp40). These PPIase proteins are indispensable for the regulation of the HSP90 conformational cycle and chaperone activity [22]. As determined by HSP90α C-terminal inhibitor assay, SL-145 competitively inhibited the binding ability of the co-chaperone PPID via direct interaction between SL-145 and the C-terminal domain of HSP90 (Fig. 1J). We further demonstrated that SL-145 had no binding affinity to N-terminal HSP90α (Fig. 1K). Docking studies revealed that SL-145 fits comfortably within the ATP-binding pocket of hHSP90, located at the center of the interface between chain A and B (Fig. 1L). The cyclopropane carboxy group interacts with the hydrophobic surface of chain A (Fig. 1M), where the cyclopropane ring forms a hydrophobic interaction with Ala616 (Fig. 1N). The side chain NH of Lys615 (A) forms a hydrogen bond with the oxygen atom of the cyclopropane carboxy group of SL-145, forming a cation-π interaction with the benzene ring of the chromene moiety. In terms of EC surfaces and scores, the score for SL-145 at 0.196 was slightly higher than that for ATP at 0.189, indicating that SL-145 can bind to the active site of hHSP90 in an ATP-competitive manner (Fig. 1O). Our in silico docking model suggests that compound SL-145 binds to the interface of HSP90 homodimer and locks open the conformation of the HSP90 homodimer, thereby inhibiting N-terminal dimerization and formation of an ATP-binding pocket.

### SL-145 eradicates BCSC-like properties in TNBC in vitro and in vivo

**Fig. 2:**
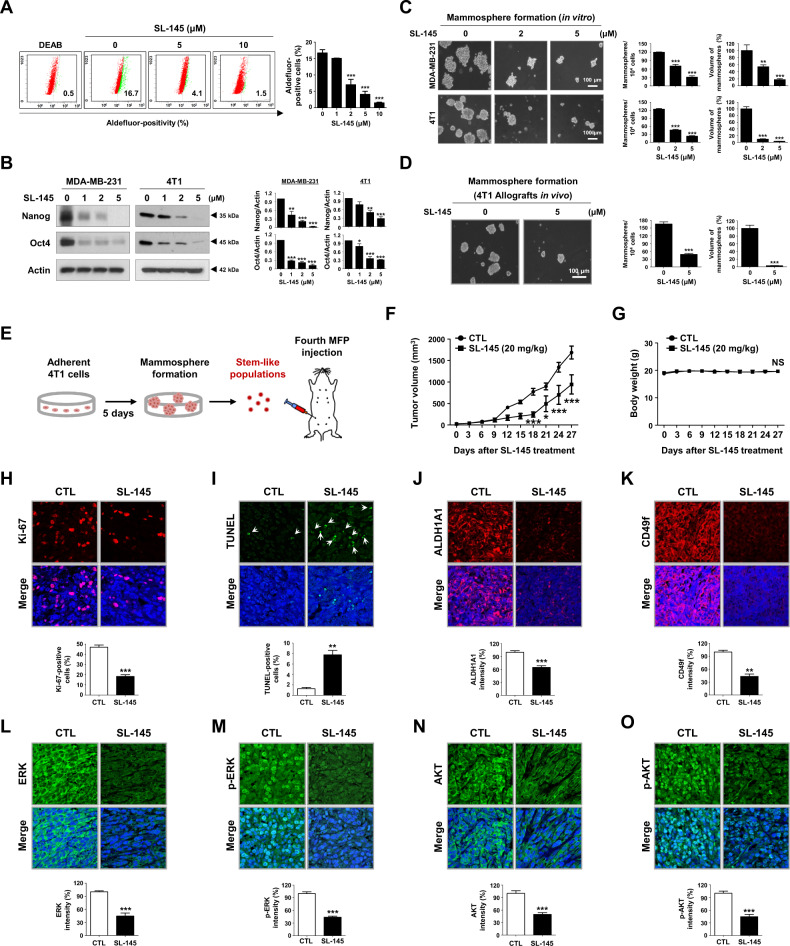
SL-145 impedes tumor growth by targeting BCSC-like properties. **A** Influence of SL-145 on ALDH1 activity in MDA-MB-231 cells. Quantitative graph of percentages of Aldefluor-positivity (right panel, ***p < 0.001, *n* = 3). **B** Changes in the expression of Nanog and Oct4 protein as determined by immunoblotting. Quantitative graphs represent the ratio of Nanog/actin and Oct4/actin in the presence or absence of SL-145 (right panel, *p < 0.05, **p < 0.01, ***p < 0.001, *n* = 3). **C** Effect of SL-145 on mammosphere formation in vitro. MDA-MB-231 (1 × 10^5^ cells/mL) and 4T1 cells (5 × 10^4^ cells/mL) were cultured in ultralow attachment plates in the presence or absence of SL-145 (2–5 μM) for 4 days. The number and volume of mammospheres were quantified by optical microscopy (**p < 0.01, ***p < 0.001, *n* = 3). **D** Effect of SL-145 on mammosphere formation in vivo. Dissociated single cells (1 × 10^5^ cells/mL) from 4T1 allograft tumors (300–350 mm^3^) were plated in ultralow attachment dishes and cultured in the presence or absence of SL-145 (5 μM) for 4 days (***p < 0.001, n = 3). **E–G** SL-145 impairs BCSC-enriched tumor growth. **E** 8 × 10^4^ cells from 4T1 mammosphere cultures were orthotopically injected into the duct of the fourth mammary gland of BALB/c female mice. Mice were administered intraperitoneally with SL-145 (20 mg/kg, every other day) or control solvent for 27 days (*n* = 8/each group). Following exposure to SL-145 or control vehicle in allografted mice, tumor growth (**F**, *p < 0.05, ***p < 0.001, *n* = 8) and body weight (**G**, NS, not significant) were evaluated. **H**–**I** Influence of SL-145 on Ki-67 expression and apoptosis in vivo. Tissue sections were immunostained for Ki-67 (**H**, red) with DAPI (blue), and Ki-67-positive cells were counted (***p < 0.001, *n* = 4). SL-145-induced apoptosis was measured by TUNEL assay (**I**, green) and nuclei were counterstained with DAPI (blue). The graph shows the percentage of TUNEL-positive cells (**p < 0.01, *n* = 4). Original magnification: × 500. **J**–**K** SL-145 administration resulted in significant downregulation of BCSC markers in vivo, as determined by immunostaining of ALDH1A1 (**J**, ***p < 0.001, *n* = 4) and CD49f (**K**, **p < 0.01, *n* = 4), respectively. **L**–**O** Effect of SL-145 on ERK, p-ERK, AKT and p-AKT expression in vivo. Tumor tissue sections were immunostained for ERK (**L**), p-ERK (**M**), AKT (**N**) or p-AKT (**O**) with DAPI (blue). Quantitative graphs of signal intensities shown in the bottom panels (***p < 0.001, *n* = 4). All images were taken with a confocal microscope (original magnification: × 500). The fluorescence intensities were analyzed using a histogram tool in the Carl Zeiss software package. Data were analyzed by Student’s *t*-test, one- or two-way ANOVA followed by Bonferroni’s post hoc test.

The loss of CSC-like characteristics leads to attenuation of epithelial to mesenchymal transition and invasion in TNBC, contributing to a decline in tumor metastasis to distant organs [23–25]. SL-145 suppressed various characteristics associated with cancer stem cells, including aldehyde dehydrogenase 1 (ALDH1) activity (Fig. 2A and Supplementary Fig. S8A), CD44^high^/CD24^low^ proliferation (Supplementary Fig. S8B), and mammosphere-forming ability (Fig. 2C). Mammospheres are tumor masses enriched with stem/progenitor cells that harbor self-renewal capacity and higher ALDH1 activity [24, 26]. Of particular note is that SL-145 predominantly suppressed the formation of mammospheres derived from 4T1 allografted tumors in vivo (Fig. 2D). The pluripotency transcription factors Nanog and Oct4 are also HSP90 clients and were significantly downregulated by SL-145 treatment (Fig. 2B).

To investigate the physiological relevance of our in vitro observations, the impact of SL-145 on tumor growth and metastasis was examined in an orthotopic allograft tumor model with BCSC-enriched 4T1 cells in vivo (Fig. 2E). SL-145 attenuated tumor growth (Fig. 2F), concomitant with a significant decrease in Ki-67 index and an increase in apoptosis (Fig. 2H, I, and Supplementary Fig. S9), without changes to the body weight of the mice (Fig. 2G). In support of the in vitro CSC-eradication observed earlier, SL-145-treated groups exhibited remarkable reductions in ALDH1A1 and CD49f expression (Fig. 2J, K and Supplementary Fig. S10). Consistent with the in vitro observations, SL-145 administration caused significant downregulation of the expression of both ERK and AKT as well as subsequent suppression of their phosphorylation (Fig. 2L–O and Supplementary Fig. S11). Overall, these findings suggest that SL-145 not only targets proliferating tumor cells via the suppression of pro-survival pathway including ERK/MEK/AKT signaling, but also abrogates BCSC-like properties, implying that SL-145 may be effective in eradicating both the tumor bulk cells and BCSC subpopulation.

### SL-145 impairs metastatic spread from primary tumors via dysregulation of STAT3 signaling

The signal transducer and activator of transcription 3 (STAT3) signaling pathway is frequently implicated in tumor progression, influencing BCSC-like traits, as well as invasiveness, angiogenesis, and metastasis [27, 28]. Breast cancer stem-enriched populations exhibited enhanced metastasis with higher STAT3 activation, whereas blockade of STAT3 reduced CSC-load and tumor sphere-forming ability, which contributes to impairment of tumorigenic potential and tumor propagation [24, 29]. STAT3 is another major client protein of HSP90, and its function is regulated by HSP90 [9, 30], making it an attractive therapeutic target.

**Fig. 3:**
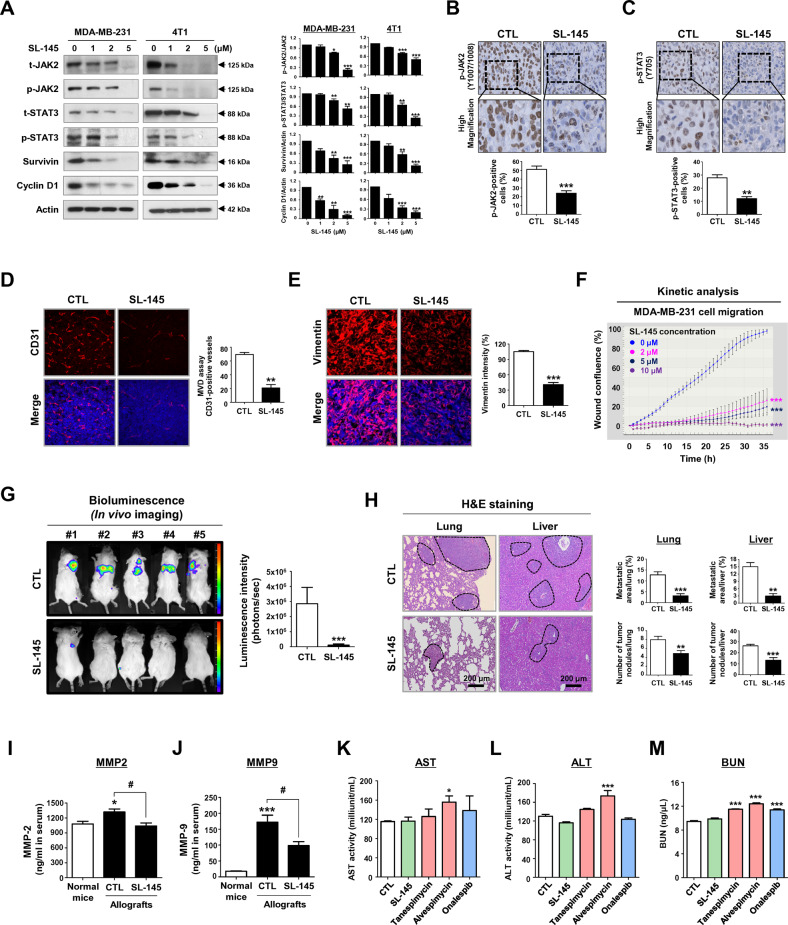
SL-145 suppresses metastases via the dysregulation of JAK2/STAT3 signaling pathway. **A** Immunoblot analyses of JAK2, phospho-JAK2 (Tyr1007/1008), STAT3, phospho-STAT3 (Tyr705), survivin, and cyclin D1 protein levels in MDA-MB-231 and 4T1 cells after treatment with SL-145 (0–5 µM, 72 h). Quantitative graphs of these protein levels shown in the right panels (*p < 0.05, **p < 0.01; ***p < 0.001, *n* = 3). Actin was used as a loading control. **B**–**C** Effect of SL-145 on the expression of phospho-JAK2 and phospho-STAT3 in allografts derived from 4T1 mammospheres. Quantitative graphs of the number of phospho-JAK2-positive cells (**B**, ***p < 0.001, *n* = 4) and phospho-STAT3-positive cells (**C**, **p < 0.01, *n* = 4) are shown in the bottom panels, respectively. **D** SL-145 administration resulted in a significant reduction in microvessel density (MVD, **p < 0.01, *n* = 4). Tumor tissues were immunostained with a specific endothelial marker CD31 (red) with DAPI (blue). Original magnification: × 200. **E** SL-145 markedly downregulates the expression of vimentin in 4T1 allografts (***p < 0.001, *n* = 4). Tumor tissue sections were immunostained for vimentin (red) with DAPI (blue). Original magnification: × 500. **F** Impact of SL-145 on cell migration of MDA-MB-231 cells. Following exposure to SL-145 (0-10 µM), kinetic analysis of cell migration was conducted using an IncuCyte™ Live-Cell Imaging System for the indicated time durations. Kinetic graph of cell migration represents the relative wound density (***p < 0.001, *n* = 8). **G** Representative bio-luminescence imaging (BLI) of metastases is shown for control and SL-145-treated groups. SL-145-treated syngeneic mice exhibited a dramatic decrease in bioluminescence signal intensity (***p < 0.001, *n* = 5). **H** H&E staining images of lung and liver sections from control and SL-145-treated mice. The black dotted areas indicate metastatic lesions in the lungs and liver. The number of tumor nodules and metastatic areas in lungs (**p < 0.01, *n* = 5) and liver (**p < 0.01, *n* = 5) was quantified. **I**–**J** Impact of SL-145 on MMP-2 and MMP-9 levels in vivo. Serum was extracted from the CTL- and SL-145-treated mice and MMP-2 (**I**)/ MMP-9 (**J**) expression was determined by ELISA assay. Serum from normal mice was used as a negative control. Data were analyzed by Student’s *t*-test (*p < 0.05, normal mouse vs control allografts; #*p* < 0.05, control allografts vs SL-145-treated allografts, *n* = 8). **K–M** Effects of SL-145, tanespimycin (17-AAG), alvespimycin (17-DMAG), and onalespib (AT13387) on serum biochemical parameters of liver and kidney toxicity. Balb/c mice (*n* = 6/each group) were intraperitoneally administered with SL-145, tanespimycin, alvespimycin, or onalespib (20 mg/kg) or control vehicle every other day for 31 days. Hepatic and renal function were assessed via ALT/AST activity and BUN concentrations in the animal serum (*p < 0.05, ***p < 0.001, *n* = 6). [AST, aspartate aminotransferase; ALT, alanine aminotransferase; BUN, blood urea nitrogen].

After exposure to SL-145, a notable reduction in the levels of phospho-JAK2 and phospho-STAT3 (Tyr705) was observed in TNBC cells (Fig. 3A), and the STAT3 dysregulation was accompanied by downregulation of its downstream targets, survivin, and cyclin D1, in both mRNA abundance and protein content (Fig. 3A and Supplementary Fig. S12). Consistent with these in vitro findings, phosphorylation of JAK2 and STAT3 was significantly reduced in allograft tumors after SL-145 administration in vivo (Fig. 3B, C and Supplementary Fig. S13).

We next evaluated the influence of SL-145 on angiogenesis and metastasis in BCSC-enriched allografts in vivo. SL-145 administration resulted in a significant reduction in intra-tumoral angiogenesis, as evidenced by reductions in CD31-positive microvessels (Fig. 3D and Supplementary Fig. S14). Since angiogenesis plays an important role in early tumor progression, the reason for the initial response of primary tumors to SL-145 may be due to its antiangiogenic inhibitory properties. Metastasis progression arises from a series of events that allow cancer stem cells to escape from the primary site, survive in the blood vessels, extravasate, and colonize at distant sites [31]. SL-145 treatment suppressed lung and liver metastases, as evidenced by significant reductions in bioluminescence intensity (Fig. 3G) and numbers of metastatic nodules in the lungs and liver (Fig. 3H). SL-145 potentially inhibits early colonization of tumor-initiating cells in distant organs via impairment of BCSC-like properties, suggesting that this may eventually lead to the suppression of lung and liver metastases. Since vimentin is implicated in cell motility, its downregulation by SL-145 (Fig. 3E and Supplementary Fig. S15) may help attenuate tumor cell invasion. Kinetic analysis for cell migration further supported the notion that SL-145 hampers the migratory capacity of TNBC cells in vitro (Fig. 3F). Similar to our results, Subramanian and colleagues developed the novobiocin derivatives KU711 and KU758, potent novel C-terminal inhibitors. KU711 and KU758 target tumor-initiating cells and inhibit the epithelial to mesenchymal transition and cell migratory ability as well as suppression of cancer stem-like properties for TNBC in vitro and in vivo [32].

The matrix metalloproteinases MMP-2/MMP-9 are STAT3 substrates that facilitate angiogenesis and metastasis in breast cancer [27, 33, 34]. HSP90 confers activation of MMP-2/MMP-9, which promote angiogenesis, invasion, and propagation via degradation of extracellular matrix (ECM) components and the basement membrane [35, 36]. During metastatic progression, MMP-2 and MMP-9 serum levels were significantly elevated in metastatic control mice, while this expression was significantly decreased in the presence of SL-145 treatment (Fig. 3I, J, and Supplementary Fig. S16). The interruption of STAT3 signaling by SL-145 was accompanied by a reduction in circulating MMP-2 and MMP-9 levels in the blood.

For comparison of SL-145 and geldanamycin on in vivo toxicity, vehicle, SL-145 (20 mg/kg BW) or geldanamycin (20 mg/kg BW) were intraperitoneally administered to Balb/c mice (*n* = 9/each group) for 10 days. Toxicity-related mortality was observed in the geldanamycin-treated group on days 2–4 (*n* = 9), whereas no dead animals were observed in the SL-145-treated group (Supplementary Fig. S17). We have further examined the effects of SL-145 on the hepatic and renal function, in comparison to N-terminal HSP90 inhibitors including geldanamycin derivatives such as tanespimycin (17-AAG) and alvespimycin (17-DMAG) as well as the second-generation HSP90 inhibitor onalespib (AT13387) that binds to the ATP-binding domain at the N-terminus of HSP90 in vivo. Blood biochemical analysis revealed that the hepatotoxicity indicators, ALT and AST, were significantly elevated in the presence of the first-generation HSP90 inhibitor alvespimycin (Fig. 3K, L) in agreement with clinical observations [37, 38]. Furthermore, significant kidney toxicity was observed for both the first-generation tanespimycin- and alvespimycin- as well as the second-generation onalespib (Fig. 3M). Renal dysfunction in clinical trials with tanespimycin, alvespimycin, and onalespib has been reported [38–41]. Importantly, SL-145 administration did not affect liver or kidney health, evidenced by no increase in the activity of AST, ALT, or BUN (Fig. 3K–M).

Numerous preclinical and clinical studies have been reported on inappropriate molecular targeting of N-terminal HSP90 inhibitors, such as geldanamycin and its derivatives [42]. There is little research or information on the off-target effects of inhibitors targeting the C-terminal domain of HSP90. However, most small molecule drugs have an off-target effect, binding to at least 6-11 other proteins than their intended pharmacological target, which potentially causes undesirable effects such as toxicity [43–45]. Further profiling of this novel compound targeting the C-terminal domain of HSP90 is warranted.

**Fig. 4:**
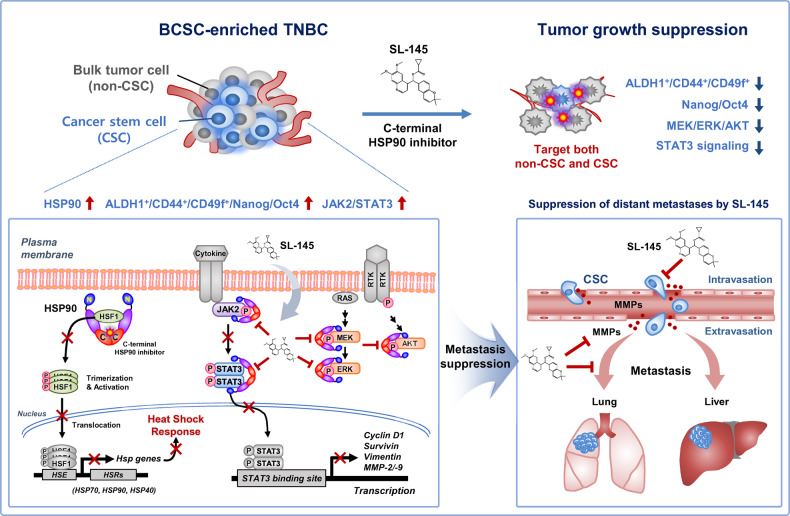
Hypothetical model illustrating the multiple actions of SL-145 on TNBC tumor growth and metastasis. Our findings indicate that (i) SL-145 targets the C-terminal domain of HSP90 without induction of the heat shock response, and (ii) effectively induces apoptotic cell death in TNBC cells. (iii) SL-145 not only kills the rapidly proliferating cells that comprise the tumor bulk, but also effectively eradicates BCSC-like traits. (iv) SL-145 administration retards tumor growth and angiogenesis, and subsequently suppresses metastasis to the lungs and liver, concomitant with suppression of the STAT3 downstream factors cyclin D1, survivin, vimentin, and MMP-2/-9. (v) These phenomena are associated with an impediment of major HSP90 client oncoproteins including AKT, MEK/ERK, and JAK2/STAT3, as well as pluripotent transcription factors. Taken together, these findings support the notion that further investigation of the novel C-terminal HSP90 inhibitor SL-145 for the treatment of metastatic triple-negative breast cancers is warranted. [Heat shock response, HSR; Heat shock factor-1, HSF-1, Heat shock element, HSE].

Our findings indicate that the novel C-terminal inhibitor SL-145 elicits anti-tumor and anti-metastatic effects without triggering the HSR due to its unconventional targeting of the C-terminal region. SL-145 effectively targets BCSC-like traits, accompanied by a significant reduction in CD44, CD49f and ALDH1 expression, as well as impairment of mammosphere-forming ability. SL-145 simultaneously inhibits multiple oncogenic signaling pathways including AKT, MEK/ERK, and JAK2/STAT3 in vitro and in vivo. It is noteworthy that SL-145 did not affect markers of hepatic and renal acute toxicity and had less cytotoxicity in non-malignant cells. SL-145 may therefore have potential to address current limitations in the treatment of TNBC (Fig. 4, hypothetical model).

## Supplementary information

Supplementary Materials and Methods

Supplementary Figures and Legends (Fig. S1-S17)

Supplementary Information_Synthesis and Purity of SL-145
