# Supplementary material for: A novel HSP90 inhibitor SL-145 suppresses metastatic triple-negative breast cancer without triggering the heat shock response
Source: Oncogene. 2022 May 2;41(23):3289–97. doi: 10.1038/s41388-022-02269-y (PMC9166677; doi:10.1038/s41388-022-02269-y)
Supplement: Supplementary file 1 — Supplementary Materials and Methods [file 41388_2022_2269_MOESM1_ESM.docx]

**Supplementary information**

**Supplementary Materials and Methods**

***1. Reagents, materials and antibodies***

Z-VAD-fmk, Triton X-100, propidium iodide (PI), corn oil and dimethyl sulfoxide (DMSO) were purchased from Sigma-Aldrich (St. Louis, MO). Tanespimycin, alvespimycin and onalespib were purchased from Selleckchem (Houston, TX). Phosphatase inhibitor and protease inhibitor cocktail tablets were obtained from Roche Applied Sciences (Penzberg, GER). Primary antibodies used targeted the following proteins: STAT3, Ki-67, CD31, ALDH1A1 (Abcam, MA); anti-AKT, phospho-AKT (Ser473), MEK, phospho-MEK (Ser217/221), ERK, phospho-ERK (Thr202/Tyr204), JAK2, phospho-JAK2 (Tyr1007/1008), phospho-STAT3 (Tyr705), CD49f, vimentin, Nanog, Oct4 (Cell Signaling, CA); survivin, cyclin D1, HSP70, HSP90 and HSF-1 (Santa Cruz Biotechnology, Santa Cruz ,CA); β-actin (Sigma-Aldrich, Saint Louis, MO). Secondary antibodies were horseradish peroxidase (HRP)-conjugated anti-rabbit and mouse IgG (Bio-Rad Laboratories, CA); and Alexa Fluor-488 and -594 goat anti-mouse IgG (Invitrogen, Carlsbad, CA).

***2. Breast cancer cell culture***

The TNBC cell lines MDA-MB-231 (PerkinElmer, Inc. CT), Hs578T (American Type Culture Collection, ATCC), BT549 and 4T1-Luc (Japanese Collection of Research Bioresources Cell Bank, JCRB), the normal murine mammary gland epithelial cell line NMuMG (ATCC) and the normal human embryonic kidney cell line HEK293 (JCRB) were cultured in MEM or RPMI 1640 (Gibco, MD) containing 10% fetal bovine serum (FBS), and streptomycin-penicillin (100 U/ml). Normal human mammary epithelial MCF10A (ATCC) cells were cultured in Mammary Epithelial Cell Growth Medium (MEGM), including hEGF, insulin, hydrocortisone and bovine pituitary extract (SingleQuotsTM Kit, Lonza, CA) containing streptomycin-penicillin (100 U/ml). Cells were incubated at 37°C in an atmosphere of 5% CO2. All cell lines were authenticated by short tandem repeat (STR) profiling by Macrogen Inc (Seoul, South Korea).

***3. Cell viability assay***

Cell viability was assessed using a CellTiter 96* Aqueous One Solution Cell Proliferation Assay [MTS, 3-(4,5-dimethylthiazol-2-yl)-5-(3-carboxymethoxyphenyl)-2-(4-sulfophenyl)-2H-tetrazolium] (Promega, Madison, WI, USA) according to the manufacturer’s instructions. The quantity of formazan product was determined by measuring the absorbance at 490 nm with a Spectramax Plus 384 microplate analyzer (Molecular Devices, Sunnyvale, CA, USA).

***4. Annexin V/PI assay***

Cells were stained using a FITC-conjugated Annexin V apoptosis detection kit (BD Biosciences, Franklin Lakes, NJ) according to the manufacturer’s protocol. Stained cells were analyzed by flow cytometry using a Beckman Coulter Expo (Brea, CA).

***5. Caspase-3 activity assay***

Caspase-3 activity was measured using caspase 3 colorimetric assay kits, according to the manufacturer’s instructions (Sigma-Aldrich, MO). Caspase-3 activity was analyzed by absorbance at 405 nm with a Spectramax Plus384 microplate analyzer (Molecular Devices, CA).

***6. CD44^high^/CD24^low^ staining***

CD44^high^/CD24^low^ staining was used to identify BCSC-like cells. Cells were incubated for 30 min at 4°C with FITC- and PE-conjugated anti-mouse IgG or FITC-conjugated anti-CD24 and PE-conjugated anti-CD44 antibodies (BD Biosciences) and analyzed by flow cytometry.

***7. Aldefluor-positivity assay***

An Aldefluor assay kit (Stemcell Technologies, Vancouver, BC) was used to assess ALDH1 activity. Cells were incubated for 45 min at 37°C in Aldefluor assay buffer containing the ALDH protein substrate BODIPY-aminoacetaldehyde (BAAA, 1 µM per 0.5⨯10^6^ cells). As a specific inhibitor of ALDH1, 50 mM diethylamino-benzaldehyde (DEAB) was used to define the Aldefluor-positive population with a flow cytometer.

***8. Immunoblot analysis***

Cells were solubilized in lysis buffer [30 mM NaCl, 0.5% Triton X-100, 50 mM Tris-HCl (pH 7.4)] containing phosphatase and protease inhibitor cocktail tablets. Supernatant was collected after centrifugation (14,000 g, 4°C, 20 min) and protein concentrations were measured with a Bradford protein assay kit (Bio-Rad Laboratories). Equal quantities of protein (30 μg) were subjected to SDS-PAGE and electrotransferred onto a nitrocellulose membrane (GE Healthcare Life Sciences, Buckinghamshire, UK). The membranes were incubated overnight at 4°C with primary antibodies diluted in 5% BSA [AKT (1:2000), phospho-AKT (1:2000), ERK (1:2000), phospho-ERK (1:2000), JAK2 (1:2000), phospho-JAK2 (1:2000) (Tyr1007/1008), STAT3 (1:3000), phospho-STAT3 (1:2000), cyclin D1 (1:3000), survivin (1:2000), MEK (1:3000), phospho-MEK (1:3000), HSP70 (1:3000), Nanog (1:2000), Oct4 (1:2000) or β-actin (1:5000)], followed by incubation with HRP-conjugated rabbit or mouse secondary antibody (1:3000–1:10,000). Signal intensity was detected using a Chemiluminescence Kit (Thermo Fisher Scientific Inc., Rockford, IL) on X-ray film (Agfa Healthcare, Mortsel, Belgium) and quantitated using AlphaEaseFC software (Alpha Innotech, San Leandro, CA).

***9. HSP90α (C-Terminal) inhibitor screening assay***

An HSP90α (C-Terminal) Inhibitor Screening Assay Kit (BPS Bioscience, CA) was used to evaluate inhibition of the interaction between C-terminal HSP90α and its co-chaperone peptidylprolyl isomerase D (PPID, also known as cyclophilin D) by HSP90 inhibitors, as previously described [1]. The HSP90 inhibitor (SL-145, novobiocin or geldanamycin) was added at several concentrations (100-1000 µM) to the reaction buffer containing both diluted HSP90α (1.5 ng/µl) and PPID (10 ng/µl) in an Optiplate-384 (PerkinElmer, Inc. USA), and then reacted with Detection AlphaLISA® Acceptor Beads and streptavidin-conjugated donor beads (PerkinElmer, Inc. USA). The C-terminal HSP90α:PPID binding activity was analyzed using an AlphaScreen® microplate reader (Varioskan LUX™, Thermo Fisher Scientific, Rockford, IL).

***10. N-terminal HSP90 binding activity assay***

For the N-terminal HSP90 binding activity assay, an HSP90α N-Terminal domain Assay Kit (BPS Bioscience, CA) was used according to the manufacturer’s protocol. This assay is a competitive binding assay based on the binding of fluorescently labeled geldanamycin, an N-terminal HSP90 inhibitor, to purified recombinant HSP90α. Briefly, SL-145, novobiocin, or geldanamycin (0-1000 nM) dissolved in DMSO were incubated with the reaction mixture containing both FITC-labeled geldanamycin (100 nM) and HSP90α (17 ng/µl) for 3 h at room temperature. The N-terminal HSP90 binding activity was determined by fluorometric detection (λex 485nm, λem 530 nm) using a fluorescence microplate reader (SpectraMax Gemini EM, Molecular Devices, San Jose, CA).

***11. Chromatin immunoprecipitation (ChIP) assay***

Chromatin immunoprecipitation (ChIP) was performed using Dynabeads Protein A and G (Thermo Fisher Scientific, Inc.). Cells (1.0 x 10^7^) were cross-linked with 1% formaldehyde for 10 min at 25 ˚C with continued agitation and the crosslinking reaction was stopped by adding glycine to a final concentration of 0.125 M for 5 min at 25 ˚C with continued agitation. Cells were then resuspended and lysed for 10 min at 4 ˚C with ChIP lysis buffer. The lysate was sonicated using an ultrasonicator (SONICS, Inc.). Fragment sizes were between 250-750 bp. Samples were diluted with ChIP lysis buffer and pre-cleared with 50 µl of Dynabeads Protein A and G for 1 h at 4 ˚C. Primary antibodies were added to the pre-cleared supernatants, before the mixtures were incubated overnight at 4 ˚C. HSF-1 antibody (1:100 dilution) and IgG antibody (1:1,000 dilution) were used for ChIP analysis. Next, 50 µl Dynabeads Protein A and G were added to the samples, and the mixtures were incubated for 2 h at 4 ˚C. The beads were subsequently washed with wash buffer (low-salt RIPA, high-salt RIPA, LiCl and TE). Precipitated chromatin was eluted in 100 µl elution buffer (0.1 M NaHCO3 and 1 % SDS) for at least 15 min at 65 ˚C. The chromatin was then treated with RNase A for 1 hour at 37 ˚C and proteinase K was added to each sample for 1 hour at 65 ˚C. Reverse cross linking was performed overnight at 65 ˚C, and DNA was purified using a QIAquick PCR Purification kit (Qiagen). Real-time PCR analysis was performed on DNA isolated from the immunoprecipitated protein using primers specific to the human HSP70.1 promoter [2]. The primers were used for real-time PCR of the human HSP70 gene (HSE1, -221 to -114) were the following: HSP70 forward primer, 5’-CCCTGTCCCCTCCAGTGAAT-3’ and HSP70 reverse primer 5’-ACCAATCAGAGGCCAGAGT-3’.

***12. Real-time quantitative polymerase chain reaction (RT-qPCR) analysis***

Total RNA was extracted using a RNase mini kit (Qiagen, Valencia, CA, USA). The cDNA was synthesized from total RNA using oligo-dT random primers and SuperScript^TM^ III Reverse Transcriptase (Invitrogen, Carlsbad, CA, USA) according to each manufacturer’s protocol. The following primers were used for RT-qPCR: HSP70 forward primer, 5’-CGACCTGAACAAGAGCATCA-3’ and HSP70 reverse primer, 5’-AAGATCTGCGTC-TGCTTGGT-3’, and GAPDH forward primer, 5’-GGGAGCCAAAAGGGTCATCATCTC-3’ and GAPDH reverse primer, 5’-CCATGCCAGTGAGCTTCCCGTTC-3’. The reaction volume of the RT-qPCR was 20 µL containing 10 µL of Power SYBR™ Green PCR Master Mix (Thermo Fisher Scientific, Waltham, MA, USA), 1 µL of forward primer (0.5 µM), 1 µL of reverse primer (0.5 µM), 2 µL of cDNA solution, and 6 µL dH_2_O. PCR was carried out using QuantStudio 6 Flex (Applied Biosystems, Foster City, CA, USA) with QuantStudio^TM^ Real-Time PCR Software under the following conditions: after an initial denaturation at 95ºC for 10 min, cDNA amplification was performed at 95ºC for 15 sec and 60ºC for 1 min for 40 cycles. Relative mRNA levels in the cDNA samples were calculated based on the comparative Ct method (ΔΔCt) with the normalization factor being GAPDH.

***13. Reverse transcription PCR analysis***

Total RNA was extracted using an RNeasy mini kit (Qiagen, CA), according to the manufacturer’s instructions. Amplification of transcripts was achieved by reverse transcriptase PCR using 1 μg/μL total RNA, Molony Murine Leukemia Virus reverse transcriptase (MMLV; Gibco/BRL, MD), and oligo-d(T)15 primers (Roche Applied Sciences). PCR amplification was performed using the following primers: cyclin D1, forward 5’-ATG TTC GTG GCC TCT AAG ATGA-3’, reverse 5’-CAG GTT CCA CTT GAG CTT GTTC-3’, survivin, forward 5’-AGT GAG GGA GGA AGA AGG CA-3’, reverse 5’-ATT CAC TGT GGA AGG CTC TGC-3’, MMP-2, forward 5’-TCT CCT GAC ATT GAC CTT GGC-3’, reverse 5’-CAA GGT GCT GGC TGA GTA GAT C-3’; MMP-9, forward 5’-TTG ACA GCG ACA AGA AGT GG-3’, reverse 5’-CCC TCA GTG AAG CGG TAC AT-3’. The PCR products were separated on 1.2% agarose gels and visualized using a Gel Doc™ XR+ System (Bio-Rad Laboratories).

***14. Immunocytochemistry***

Cells on 8-well chamber slides (BD Biosciences, Franklin Lakes, NJ) were fixed with 4% paraformaldehyde, washed with PBS, and incubated with 0.2% Triton X-100 for 10 min. The cells with primary antibodies in antibody-diluent (Dako, Glostrup, Denmark) were incubated overnight at 4°C, and then incubated with secondary antibodies (Alexa Fluor®-488 or -594) Carlsbad, CA). Cells were mounted with ProLong Gold Antifade Reagent with DAPI (Life Technologies, Carlsbad, CA). Images were acquired using a Carl Zeiss confocal microscope (Weimar, Germany), and the intensity of the images was analyzed using the intensity profile tool.

***15. Molecular Modeling and Docking Analysis***

Docking modeling was conducted using the Tripos Sybyl-X 2.1 program in the Windows 7 operating system, and the analysis and visualization of docking results were performed using Maestro Graphic User Interface in Schrödinger 19-4. For the electrostatic complementarity analysis of docking models, the Flare^TM^ software (version 3.0) was used. Molecular structures of ligands were prepared in mol2 format using the sketch module in Sybyl. Gasteiger-Hückel charges were assigned to all atoms of ligand, and optimized by energy minimization using the conjugate-gradient method with the convergence criterion of 0.001 kcal mol^-1^.Å^-1^. We used the Surflex-Dock program for docking modeling of the binding pose of SL-145 in the C-terminal domain of human HSP90 (hHSP90). Our previously reported docking model of the hHSP90:ATP complex was used as a receptor, and SL-145 was docked into the ATP-binding site of this model. The protomol, a computational description of the binding cavity at which putative ligands are aligned, was defined by 20 amino acid residues adjacent to ATP with a threshold parameter of 0.50 and a bloat parameter of 0 Å. Docking was conducted using the default settings of Surflex-Dock generating 50 maximum poses per ligand. The binding affinity of each pose of the ligand was estimated by Surflex-Dock score (-log K_d_) which takes into account hydrophobic, polar, repulsive, entropic and salvation terms. The final docking model was selected by visual inspection, considering the Surflex-Dock score. To calculate the electrostatic complementarity (EC) of the docked complex, the selected pose of SL-145 and hHSP90 was exported in SDF format to the Flare program, and EC surfaces and scores between ligand and receptor were generated in the default settings.

***16. Allograft in vivo experiments and bioluminescence imaging***

All animal procedures were carried out in accordance with animal care guidelines approved by the Korea University Institutional Animal Care and Use Committee (IACUC). Five-week-old female BALB/c mice were obtained from the Shizuoka Laboratory Animal Center (Shizuoka, Japan) and housed in a specific pathogen-free environment. The animals were acclimated for 1 week prior to the study and had free access to food and water. 1×10^5^ cells from 4T1 mammospheres were implanted subcutaneously in the right flank of 6-week-old BALB/c female mice (n=8/each group). When average tumor volumes reached 100 mm3, the animals were randomized into 2 groups (n=8/each group), vehicle (DMSO/corn oil, 1:9) or SL-145 (20 mg/kg/day, every other day) was administered intraperitoneally for 27 days, and tumor volumes were measured using a caliper and calculated using the formula V=(Length×Width2)/2. After 24 h of the last administration of SL-145, the animals were then anesthetized and subjected to NightOWL LB983 bioluminescence imaging (BLI) (Berthold Technologies, TN). D-luciferin sodium salt (BioVision Inc. Milpitas, CA) at 150 mg/kg body weight in 100 µl PBS was administered intraperitoneally as a substrate before imaging. The captured images were quantified (photons/sec) using the IndiGo™ software package. Kaplan-Meier survival curves were analyzed using GraphPad Prism 5.0 software (SanDiego, CA, USA). Balb/c mice (n=9/each group) were intraperitoneally administered with SL-145 (20 mg/kg), geldanamycin (20 mg/kg) or control vehicle every other day for 27 days. The physical condition of the animals was monitored twice daily to determine the incidence of death. Results are represented as mean ± SEM (n=9); the comparison of survival curve was analyzed by log-rank (Mantel-Cox) test.

***17. Immunohistochemistry and in-situ localization of apoptosis (TUNEL)***

Tumors were removed, fixed in 10% neutral-buffered formalin, and embedded in paraffin. Tissue sections of 4 μm thickness were mounted on positively charged glass slides and deparaffinized with xylene and dehydrated through a graded alcohol series to water. For antigen retrieval, sections were boiled in citric acid buffer (pH 6.0). Tissue sections with primary antibodies (Ki-67; 1:150, CD49f; 1:150, ALDH1A1; 1:150, AKT; 1:150, p-AKT; 1:100, ERK; 1:150, p-ERK; 1:100, CD31; 1:100 or vimentin; 1:150) in antibody-diluent (Dako, Glostrup, Denmark) were incubated overnight at 4°C. For secondary antibody reactions, the sections were incubated with fluorescence-conjugated secondary antibody at RT for 2 hours, followed by ProLong gold antifade reagent with DAPI (Life Technologies, CA). Phospho-JAK2 (1:100) and phospho-STAT3 (1:100) staining was performed using a Vectastain ABC kit (Vector Laboratories, CA) with diaminobenzidine (DAB, GBI labs, WA) followed by hematoxylin counterstaining (Sigma, MO) in accordance with the manufacturer's instructions. In situ TUNEL was carried out on tissue sections using a TUNEL kit (Roche Applied Sciences, Penzberg, GER) in accordance with the manufacturer’s instructions.

***18. Mammosphere formation in vitro and in vivo assays***

For the *in vitro* mammosphere-forming assay, cells were plated in ultralow attachment dishes and cultured in HuMEC basal serum-free medium (Gibco, MD), supplemented with B27 (1:50, Invitrogen), 20 ng/mL basic fibroblast growth factor (bFGF, Sigma), 20 ng/mL human epidermal growth factor (EGF, Sigma), 4 μg/ml heparin, 1% antibiotic-antimycotic, and 15μg/mL gentamycin at 37°C in an atmosphere of 5% CO2. For the in vivo assay, xenografted tumors were prepared as per the animal model described in Animals, xenograft experiments. The tumors were harvested when volumes reached 300-350 mm^3^ and were dissociated with type III collagenase (2 mg/ml) until the tissues were digested into single cells. The resultant single cells were filtered through a 40 µm cell strainer, centrifuged at 200 g for 5 min and washed with medium containing 0.2% bovine serum albumin (BSA). The cells were seeded in ultralow attachment dishes and cultured under the same conditions as for the in vitro protocol. The number and volume of the mammospheres were determined under an Olympus IX 71 inverted microscope.

***19. MMP-2 and MMP-9 ELISA assay***

Matrix metalloproteinase MMP-2 and MMP-9 levels in mouse serum were measured using ELISA kits (R&D Systems, Minneapolis, MN), according to the manufacturer’s instructions. The quantity of MMP-2 and MMP-9 was determined by measuring the absorbance at 450 nm with a Spectramax MAX 190 microplate reader (Molecular Devices, CA).

***20. Wound healing assay***

For kinetic migration analysis, MDA-MB-231 cells were seeded to ~80% confluency in 96-well plates, respectively (Essen ImageLock, Essen Biosciences, Ann Arbor, MI, USA). Wound areas were made with a 96-pin Wound Maker device and washed with PBS to prevent reattachment of dislodged cells. Cells were treated with SL-145 immediately after wound scratching, and images of the scratched fields were automatically acquired and registered every hour up to 35 h with an IncuCyte™ ZOOM® Kinetic Imaging System. The relative wound density was analyzed using the IncuCyte™ Scratch Wound Cell Migration Software Module.

***21. Serum biochemistry profiles for biomarkers of liver and renal injury***

Balb/c mice (n=6/each group) were intraperitoneally administered with SL-145, tanespimycin, alvespimycin or onalespib (20 mg/kg) or control vehicle every other day for 31 days. At sacrifice, blood samples of each animal were collected and serum samples were acquired by centrifuging at 3000 rpm for 20 min. Serum enzyme activities of aspartate aminotransferase (AST), alanine aminotransferase (ALT), and blood urea nitrogen (BUN) levels were determined with an AST, ALT and BUN assay kit following the manufacturer’s protocol (Sigma-Aldrich, Louis, MO).

***22. Statistical analysis***

All data were analyzed using GraphPad Prism 5.0 statistical software (San Diego, CA). The results are presented as mean ± SEM of at least three independent experiments. Data were analyzed by student’s *t*-test, and one- or two-way ANOVA as appropriate. Significance between multiple experimental groups was determined using the Bonferroni’s post hoc test. The comparison of survival curves was analyzed by log-rank (Mantel-Cox) test. Statistical significance was defined at *p*<0.05 (*).

**References**

1. Park JM, Kim YJ, Park S, Park M, Farrand L, Nguyen CT, et al. A novel HSP90 inhibitor targeting the C-terminal domain attenuates trastuzumab resistance in HER2-positive breast cancer. Mol Cancer. 2020;19:161.

2. Fujimoto M, Takaki E, Takii R, Tan K, Prakasam R, Hayashida N, et al. RPA assists HSF1 access to nucleosomal DNA by recruiting histone chaperone FACT. Mol Cell. 2012;48:182-94.
