# Supplementary material for: A novel HSP90 inhibitor SL-145 suppresses metastatic triple-negative breast cancer without triggering the heat shock response
Source: Oncogene. 2022 May 2;41(23):3289–97. doi: 10.1038/s41388-022-02269-y (PMC9166677; doi:10.1038/s41388-022-02269-y)
Supplement: Supplementary file 2 — Supplementary Figures and Legends (Fig. S1-S17) [file 41388_2022_2269_MOESM2_ESM.pdf]

# Supplementary information

## Supplementary Figures and Legends (Fig. S1-S17)

### *Supplementary Fig. S1*

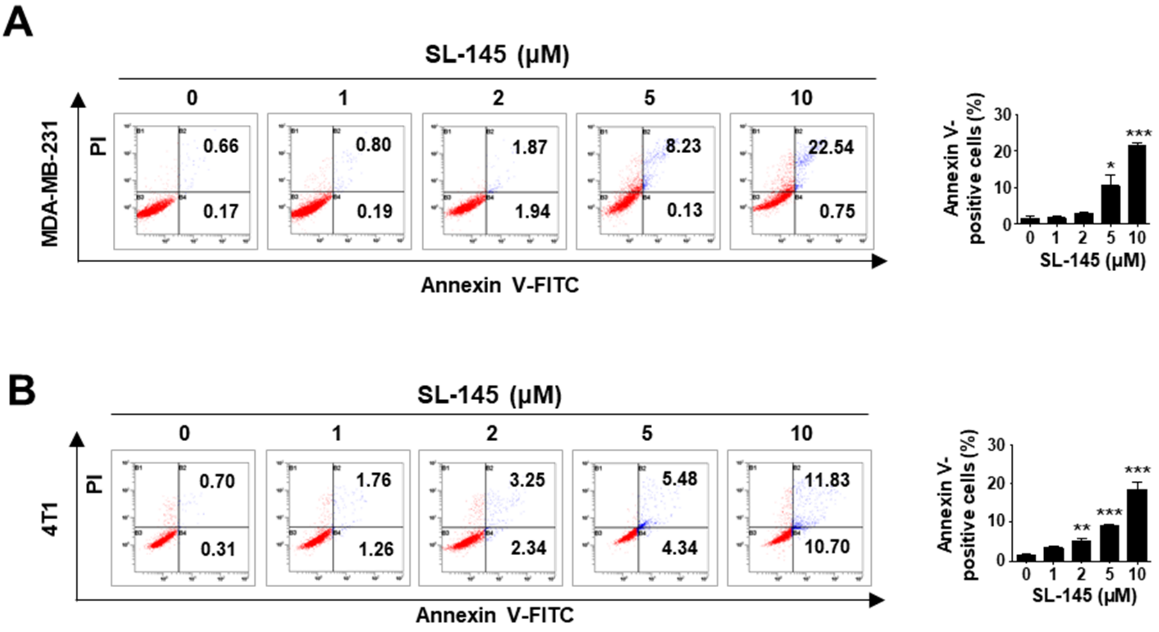


**Supplementary Fig. S1. SL-145 induces apoptosis in TNBC cells.** MDA-MB-231 (**A**) and 4T1 (**B**) cells were treated with SL-145 (0-10 µM, 72 h) and early and late apoptosis assays with annexin V/PI staining were performed with flow cytometry. The percentages of the annexin V-positive cell populations were quantified (right panel, *$p$<0.05; **$p$<0.01; ***$p$<0.001, n=3). The results are presented as mean ± SEM of at least three independent experiments analyzed by one-way ANOVA followed by Bonferroni's post hoc test.

*Supplementary Fig. S2*

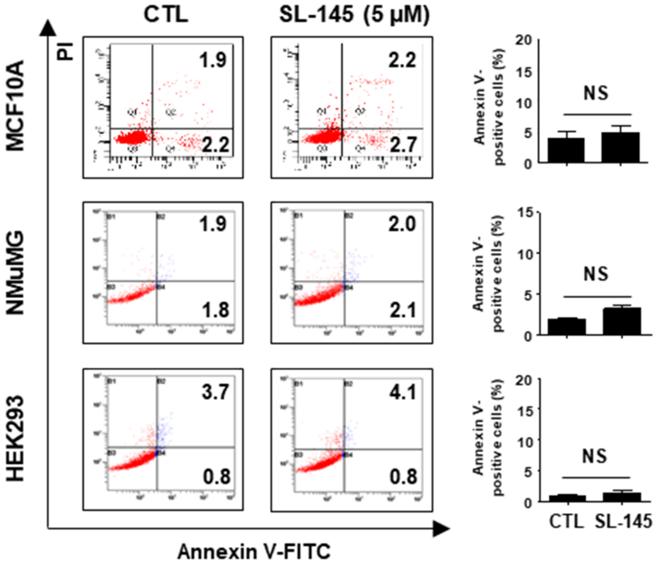


**Supplementary Fig. S2. SL-145 does not induce apoptosis in non-malignant cells.** Normal human mammary epithelial MCF10A, normal murine mammary gland NMuMG and normal human embryonic kidney HEK293 cells were treated with SL-145 (5 μM) for 72 h. Apoptosis assays with annexin V/PI staining were performed using flow cytometry, and the percentages of the annexin V-positive cells determined (NS, not significant, n=3). The results are presented as mean ± SEM of at least three independent experiments. Data were analyzed by Student's *t*-test.

## *Supplementary Fig. S3*

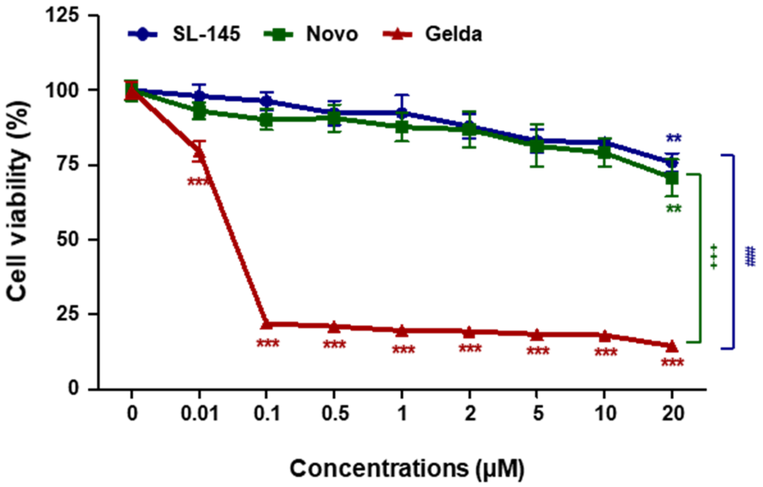


**Supplementary Fig. S3. C-terminal HSP90 inhibitors SL145 and novobiocin exhibit less cytotoxic to normal human mammary epithelial MCF10A cells.** Cells were treated with various concentrations (0.01–20 μM) of SL-145, novobiocin or geldanamycin for 72 h. Cell viability was determined by MTS assay (****p*<0.01, compared to the control; ###*p*<0.001, SL-145 vs Gelda; +++*p*<0.001; Novo vs Gelda, n=4). The results are presented as mean ± SEM of at least three independent experiments analyzed by two-way ANOVA followed by Bonferroni's post hoc test.

***Supplementary Fig. S4***

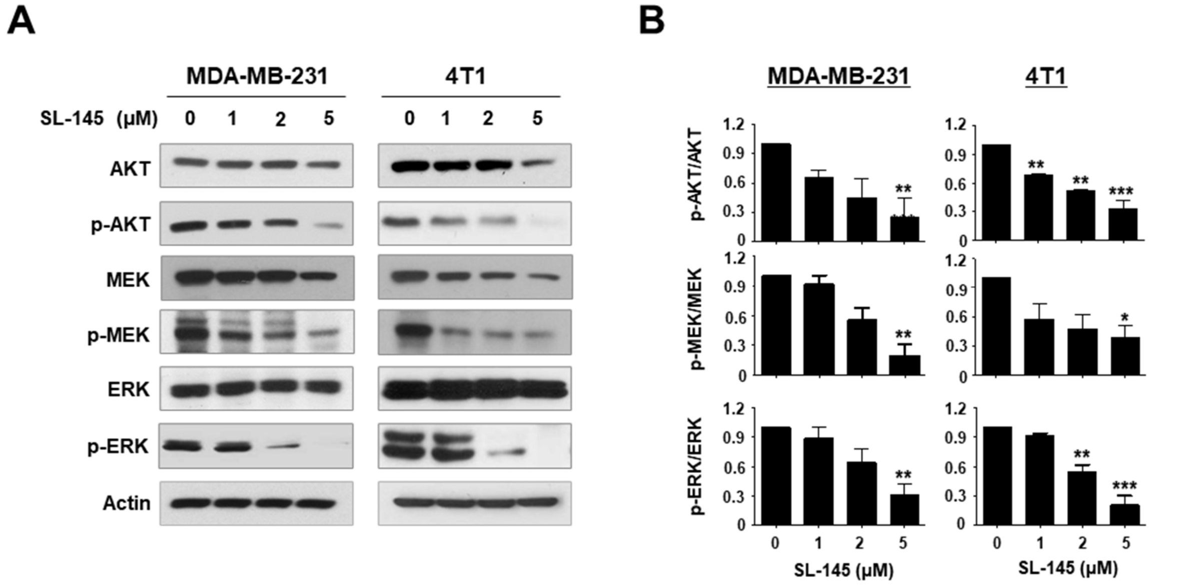


**Supplementary Fig. S4. SL-145 downregulates the expression of HSP90 client proteins.** **A** Immunoblot analyses of AKT, phospho-AKT (Ser473), MEK, phospho-MEK (Ser218/222), and ERK, phospho-ERK (Thr202/Tyr204) protein expression in MDA-MB-231 and 4T1 cells following exposure to SL-145 (1–5 μM, 72 h). Actin was used as a loading control. **B** Quantitative graphs represent the ratio of phosphorylated-/total-proteins in the presence or absence of SL-145 (**p*<0.05; ***p*<0.01; ****p*<0.001, n=3). The results are presented as mean ± SEM of at least three independent experiments analyzed by one-way ANOVA followed by Bonferroni's post hoc test.

*Supplementary Fig. S5*

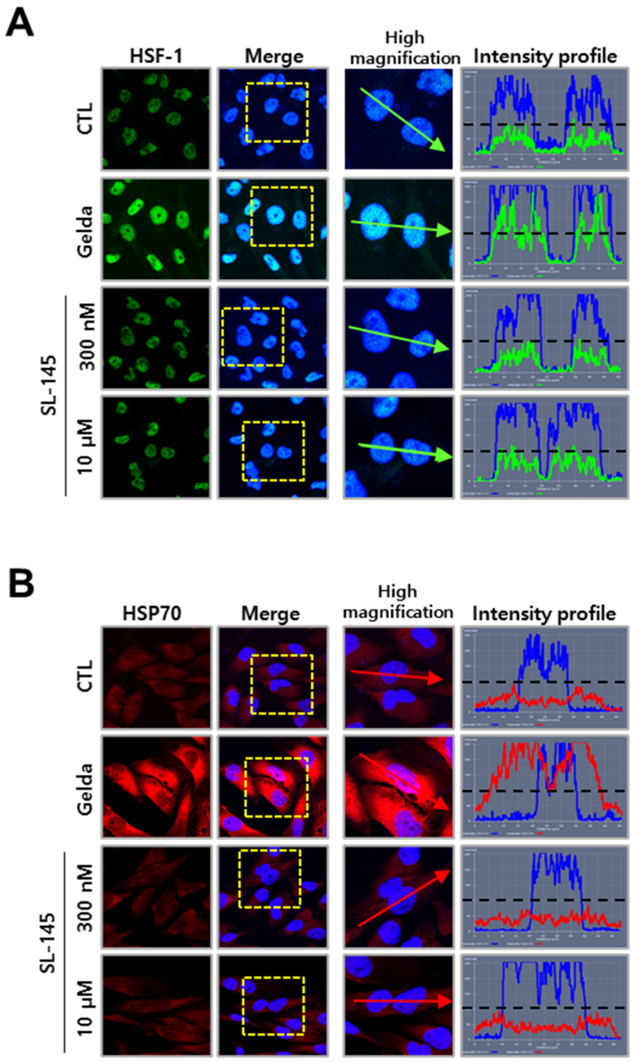


**Supplementary Fig. S5. SL-145 does not induce the heat shock response. A-B** MDA-MB-231 cells immunostained for HSF-1 (**A**, green) and HSP70 (**B,** red) with DAPI (blue) after exposure to SL-145 (300 nM and 10 µM) and geldanamycin (300 nM) for 24 h. No increase in HSF-1 or HSP70 was observed following treatment with SL-145, whereas geldanamycin upregulated HSP70 and increased nuclear accumulation of HSF-1. Intensity of nuclear HSF-1 (green) and cytosolic HSP70 (red) is represented in arbitrary units as defined by the software using the intensity profile tool. Gelda: geldanamycin.

***Supplementary Fig. S6***

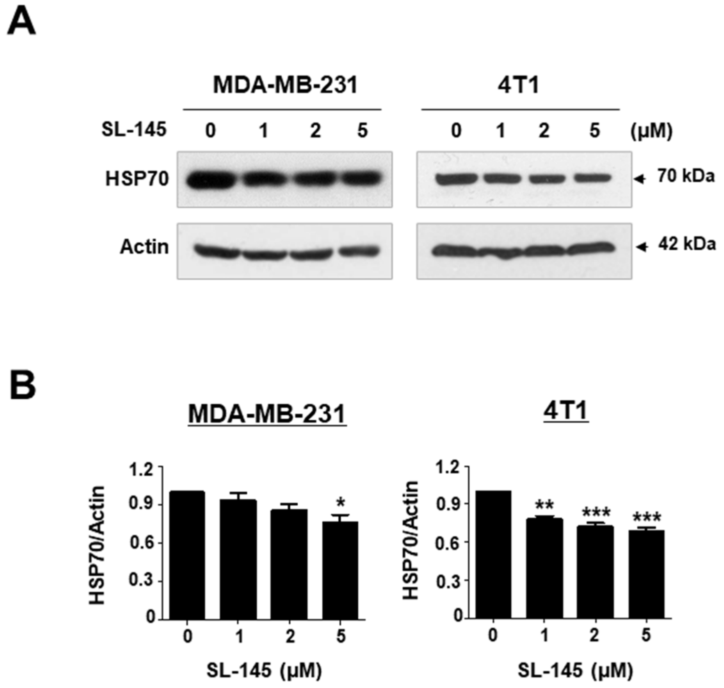


**Supplementary Fig. S6. Effects of SL-145 on expression of HSP70 protein. A** Immunoblot analyses of expression of HSP70 protein in MDA-MB-231 and 4T1 cells after exposure to SL-145 (1–5 μM, 72 h). Actin was used as a loading control. **B** Quantitative graphs represent the ratio of HSP70/actin in the presence or absence of SL-145 (bottom panel, $*p<0.05$; $**p<0.01$; $***p<0.001$, n=3). The results are presented as mean ± SEM of at least three independent experiments analyzed by one-way ANOVA followed by Bonferroni's post hoc test.

***Supplementary Fig. S7***

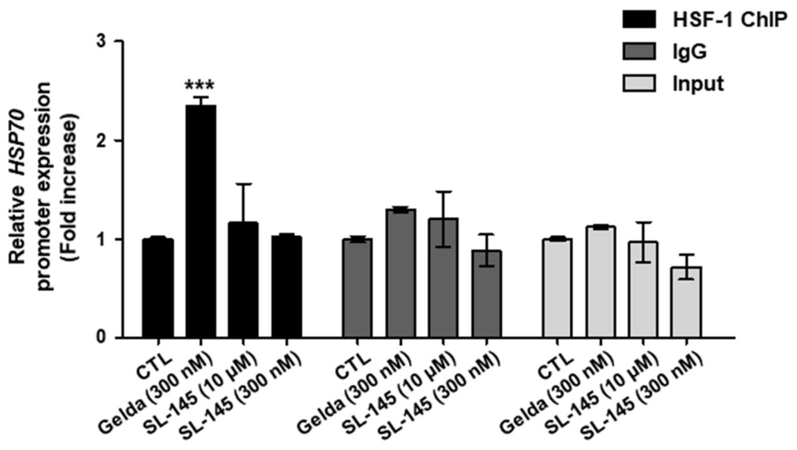


**Supplementary Fig. S7. Effect of SL-145 and geldanamycin on HSF-1 binding to the HSP70 promoter.** MDA-MB-231 cells were treated with SL-145 (300 nM and 10 μM) or geldanamycin (300 nM) for 24 h, and then analyzed for binding of HSF-1 to the HSP70 promoter by ChIP assay. ChIP-enriched DNAs using preimmune IgG or anti-HSF-1 antibody, as well as input DNAs, were prepared and DNA fragments of the HSP70 gene (HSE1, -221 to -114) were amplified by real-time PCR. The relative promoter expression was calculated as fold increase compared to the control group (***$p<0.001$, n=4). The results are presented as mean ± SEM of at least three independent experiments analyzed by one-way ANOVA followed by Bonferroni's post hoc test.

*Supplementary Fig. S8*

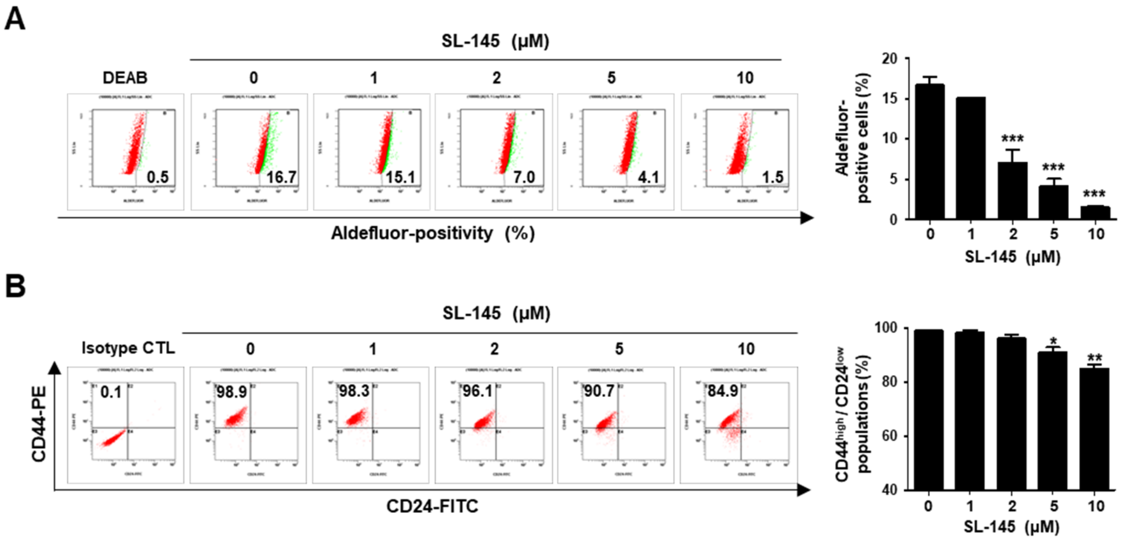


**Supplementary Fig. S8. SL-145 suppresses BCSC-like properties. A-B** Following exposure to SL-145 (1–10 μM, 72 h), ALDH1 activity and CD44$^{high}$/CD24$^{low}$ populations in MDA-MB-231 cells were evaluated by flow cytometry. The quantitative graph represents the percentage of Aldefluor-positive cells (**A**, ****p*<0.001, n=3) and CD44$^{high}$/CD24$^{low}$ populations (**B**, **p*<0.05; ***p*<0.01, n=3) are shown in the right panels. The results are presented as mean ± SEM of at least three independent experiments analyzed by one-way ANOVA followed by Bonferroni's post hoc test.

*Supplementary Fig. S9*

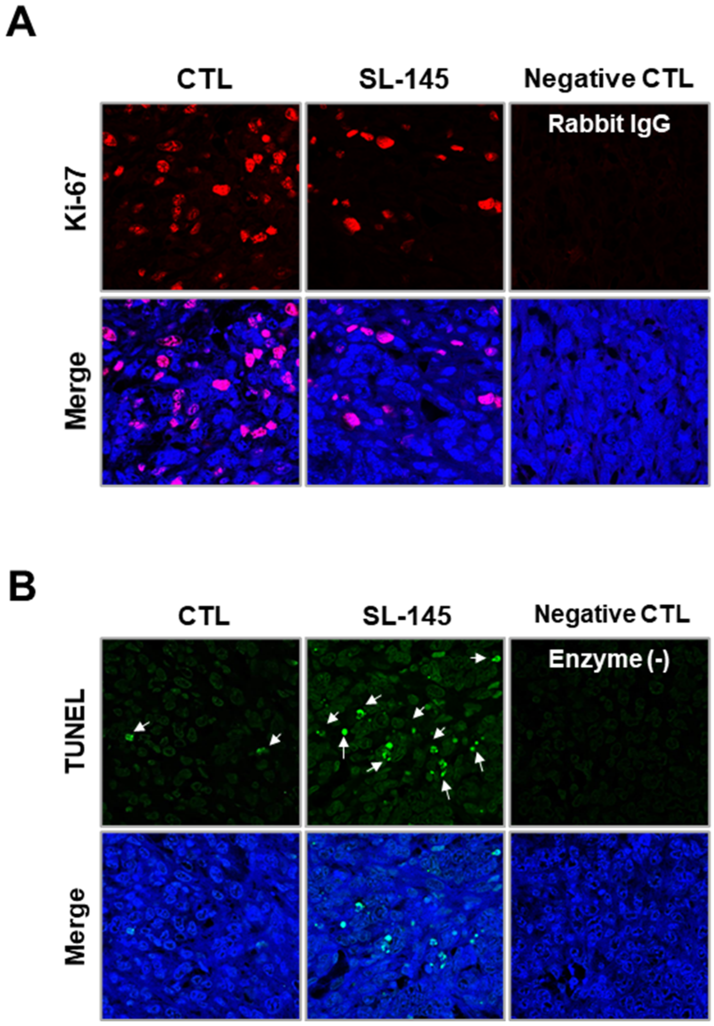


**Supplementary Fig. S9. Influence of SL-145 on Ki-67 expression and apoptosis *in vivo.* A** Effect of SL-145 on proliferating tumor cells in 4T1-allografts was examined by Ki-67 staining. Tissue sections were immunostained for Ki-67 (red) or normal rabbit IgG (as a negative control) with DAPI (blue). **B** SL-145-induced apoptosis was measured by TUNEL assay and nuclei were counterstained with DAPI (blue). Label solution without terminal transferase enzyme was used as a negative control. Original magnification: × 500.

*Supplementary Fig. S10*

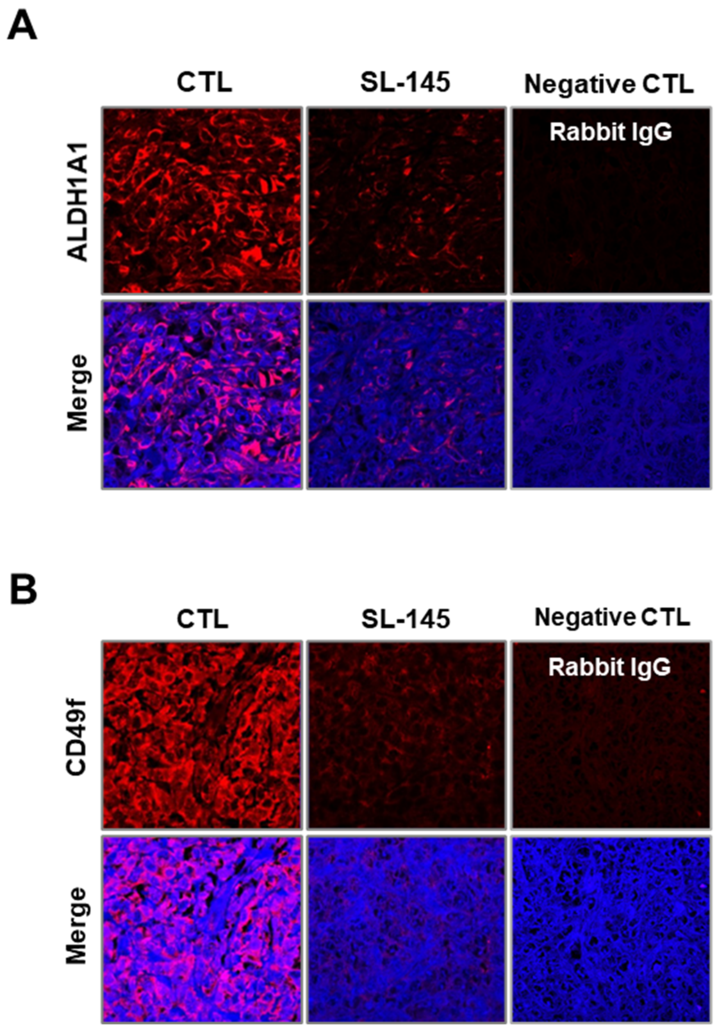


**Supplementary Fig. S10. Effect of SL-145 on ALDH1A1 and CD49f expression *in vivo.*** **A-B** SL-145 administration resulted in a significant downregulation of ALDH1A1 and CD49f in 4T1 allografts. Tumor tissue sections were immunostained for ALDH1A1 (**A**, red), CD49f (**B**, red), or normal rabbit IgG (as a negative control) with DAPI (blue). Original magnification: × 500

*Supplementary Fig. S11*

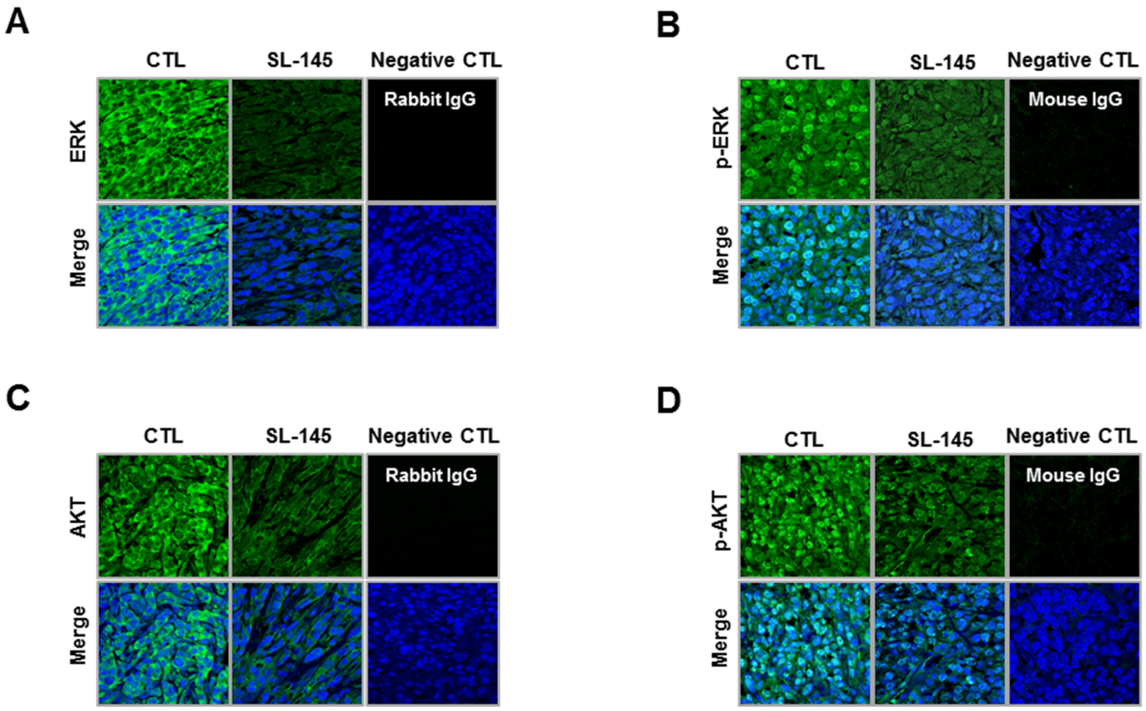


**Supplementary Fig. S11. Effect of SL-145 on ERK, p-ERK, AKT and p-AKT expression *in vivo.* A-D** SL-145 administration resulted in a significant downregulation of ERK, p-ERK, AKT and p-AKT in 4T1 allografts. Tumor tissue sections were immunostained for ERK **(A**, green), p-ERK (**B**, green), AKT (**C**, green), p-AKT (**D**, green), or normal rabbit IgG (a negative CTL) or normal mouse IgG (a negative CTL) with DAPI (blue). All images were taken with a confocal microscope (original magnification: × 500)

*Supplementary Fig. S12*

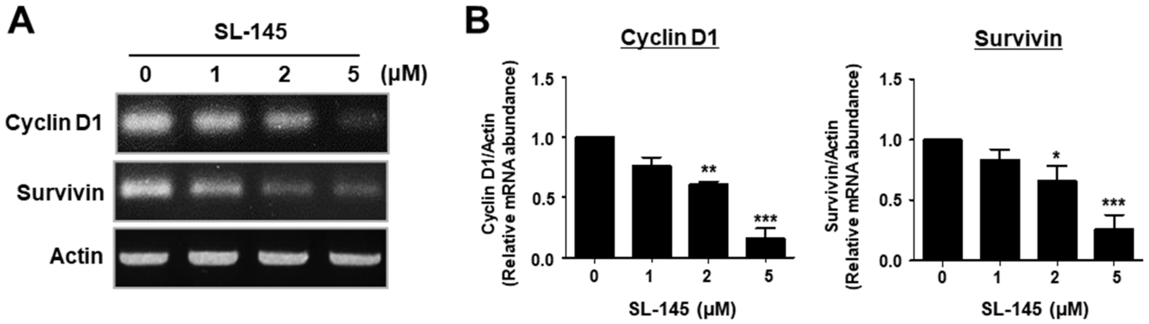


**Supplementary Fig. S12. SL-145 downregulates cyclin D1 and survivin mRNA abundance.** **A** Effect of SL-145 (1-5 μM, 72 h) on mRNA transcript levels of STAT3 downstream factors cyclin D1 and survivin in MDA-MB-231 cells. **B** Quantitative graphs of cyclin D1 (**$p<0.01$; ***$p<0.001$, n=3) and survivin (*$p<0.05$; ***$p<0.001$, n=3) mRNA abundance are shown. The results are presented as mean ± SEM of at least three independent experiments analyzed by one-way ANOVA followed by Bonferroni's post hoc test.

*Supplementary Fig. S13*

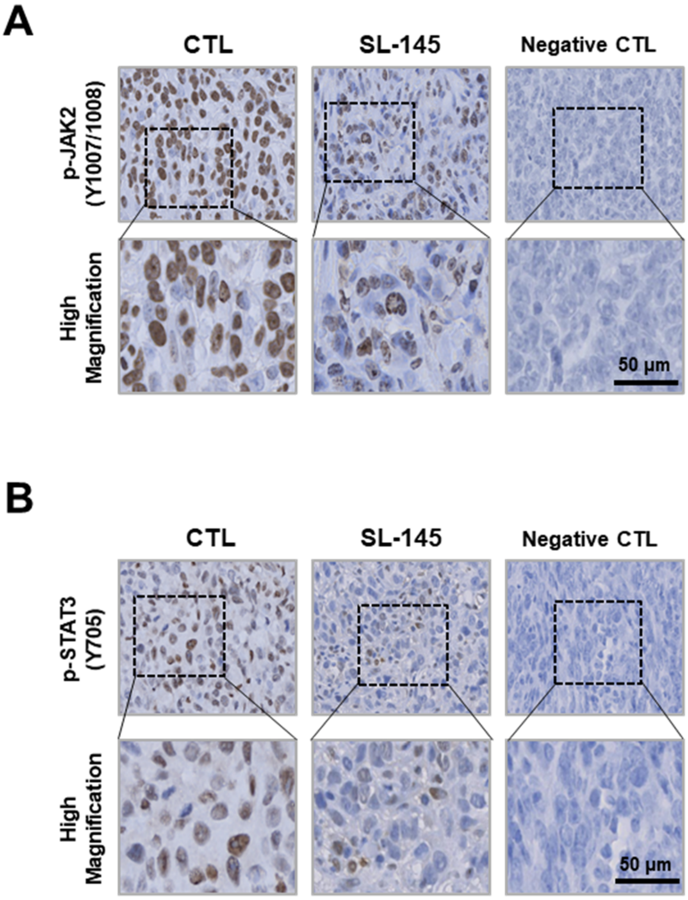


**Supplementary Fig. S13. SL-145 decreases the phosphorylation of JAK2 and STAT3 *in vivo*. A-B** Impact of SL-145 on nuclear expression of phospho-JAK2 and phospho-STAT3 in allografts derived from 4T1 mammospheres. Tumor tissue sections were immunostained with phospho-JAK2 (**A**, Tyr1007/1008), phospho-STAT3 (**B**, Tyr705) and nuclear counterstained with hematoxylin. Normal rabbit IgG was used as a negative control. Immunohistochemical microphotographs were taken using a Carl Zeiss Axio Scan.Z1, with the selected areas shown at high magnification (scale bar: 50 µm).

*Supplementary Fig. S14*

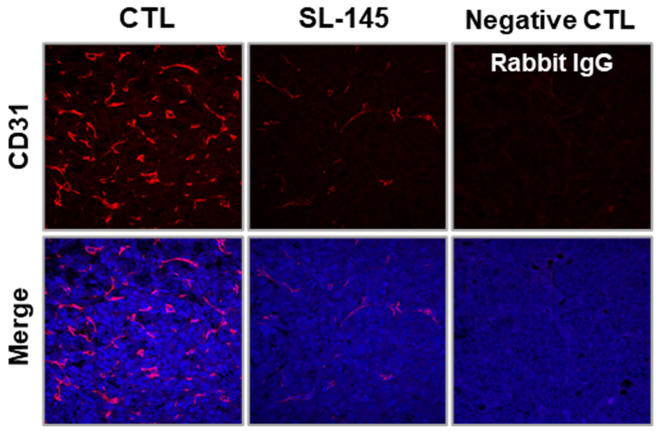


**Supplementary Fig. S14. Effect of SL-145 on tumor angiogenesis.** SL-145 administration resulted in a significant reduction in microvessel density (MVD). Tumor tissues were immunostained with a specific endothelial marker CD31 (red) or normal rabbit IgG (as a negative control) with DAPI (blue). Original magnification: × 500.

## *Supplementary Fig. S15*

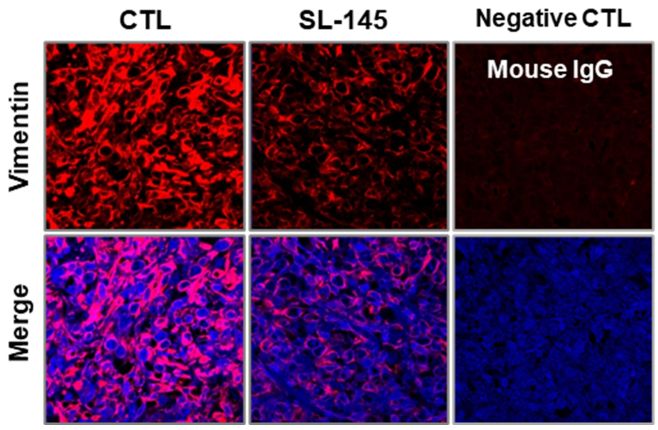


**Supplementary Fig. S15. Effect of SL-145 on vimentin expression *in vivo.*** SL-145 markedly downregulates the expression of vimentin in 4T1 allografts. Tumor tissue sections were immunostained for vimentin (red) or normal mouse IgG (as a negative control) with DAPI (blue). Original magnification: × 500.

## *Supplementary Fig. S16*

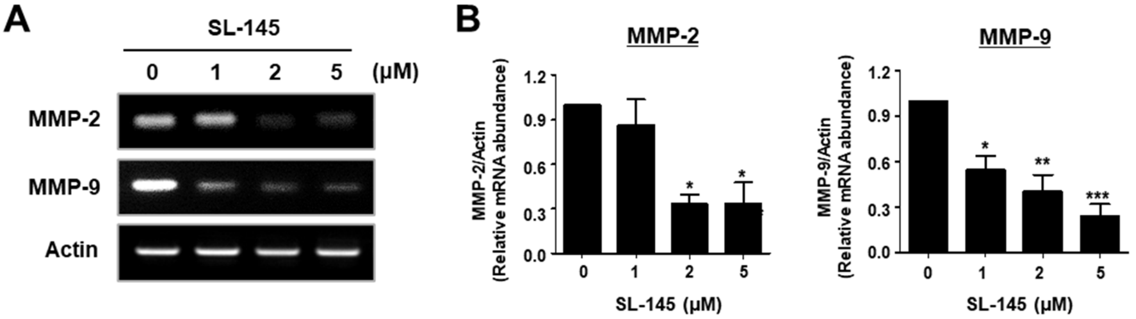


**Supplementary Fig. S16. SL-145 downregulates MMP-2 and MMP-9 mRNA abundance.** **A** Effect of SL-145 (1-5 μM, 72 h) on mRNA transcript levels of matrix metalloproteinases MMP-2 and MMP-9 in MDA-MB-231 cells. **B** Quantitative graphs of MMP-2 and MMP-9 mRNA abundance are shown in the right panel (*$p$<0.05; **$p$<0.01; ***$p$<0.001, n=3). Results are expressed as mean ± SEM; data were analyzed by one-way ANOVA followed by Bonferroni's post hoc test.

*Supplementary Fig. S17*

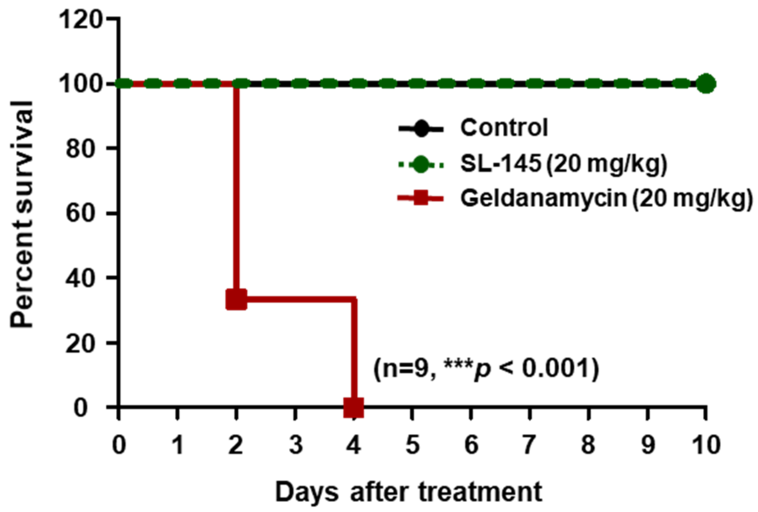


**Supplementary Fig. S17. Kaplan Meier survival analysis curves of SL-145 and Geldanamycin-treated mice.** Balb/c mice (n=9/each group) were administered intraperitoneally with SL-145 (20 mg/kg), geldanamycin (20 mg/kg) or control vehicle once daily for 10 days. Toxicity-related mortality was observed in the geldanamycin-treated group on day 2-4 (***$p$<0.001, n=9), whereas no dead animals were observed in the SL-145-treated group for 10 days. Results are expressed as mean ± SEM (n=9); the comparison of survival curves was analyzed by log-rank (Mantel-Cox) test.
