# Supplementary material for: A novel HSP90 inhibitor SL-145 suppresses metastatic triple-negative breast cancer without triggering the heat shock response
Source: Oncogene. 2022 May 2;41(23):3289–97. doi: 10.1038/s41388-022-02269-y (PMC9166677; doi:10.1038/s41388-022-02269-y)
Supplement: Supplementary file 3 — Supplementary Information_Synthesis and Purity of SL-145 [file 41388_2022_2269_MOESM3_ESM.pdf]

Supplementary information

Synthesis and purity of SL-145

1. Synthesis of SL-145

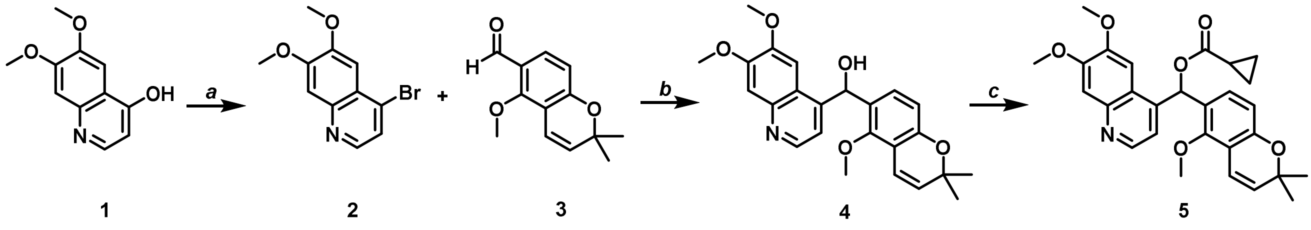

Reagents and conditions: (a) PBr_3, DMF, r.t.; (b) i) n-BuLi, THF, -78 °C, ii) **3** in THF; (c) CH_3COCl, pyridine, CH_2Cl_2, 0 °C to r.t.

General. All chemical reagents and solvents were commercially available. Melting points were determined on a melting point Buchi B540 apparatus and are uncorrected. Silica gel column chromatography was performed on a silica gel 60, 230−400 mesh, Merck. ^{1}H-NMR spectra were recorded on a JEOL JNM-LA 300 at 300 MHz. Chemical shifts are reported in ppm units with Me_4Si as a reference standard. Mass spectra were recorded on an Agilent, Q-TOF 6530 LC−MS instrument. High-performance liquid chromatography (HPLC) was performed on an Agilent 1120 Compact LC (G4288A) instrument using an Agilent TC-C18 column (4.6 mm × 250 mm, 5 μm).

4-Bromo-6,7-dimethoxyquinoline (2)

To a solution of 6,7-dimethoxyquinolin-4-ol (3.0 g, 14.62 mmol) in DMF (10 mL), PBr_3 (2.08 mL, 21.93 mmol) was added dropwise at 0 °C. The reaction mixture was allowed to slowly warm to room

temperature and stirred for 4 hrs. The reaction was cooled down to 0 °C and terminated by adding aqueous saturated $NaHCO_3$ solution to neutralize the reaction mixture. The precipitate was filtered, washed with water and diethyl ether, and dried under vacuum to afford **2** (2.42 g, 62%) as a brown solid. ^{1}H-NMR ($CDCl_3$, 300 MHz) δ 8.48 (d, J = 4.7 Hz, 1H), 7.56 (d, J = 5.0 Hz, 1H), 7.42 (s, 1H), 7.40 (s, 1H), 4.08 (s, 3H), 4.06 (s, 3H)

(6,7-Dimethoxyquinolin-4-yl)(5-methoxy-2,2-dimethyl-2H-chromen-6-yl)methanol (4)

To a solution of **2** (100 mg, 0.37 mmol) in THF (2 mL), *n*-BuLi solution in hexane (2.5 M, 0.17 mL, 0.41 mmol) was slowly added at -78 °C and stirred for 20 min. A solution of **3** (0.37 mmol) in THF (2 mL) was slowly added and stirred for 30 min. The reaction was quenched by adding saturated NH_4Cl solution, and the aqueous phase was extracted with EtOAc several times. The combined organic layers were washed with brine, dried over $MgSO_4$, filtered and the solvent was evaporated in vacuo. The residue was purified by flash column chromatography on silica gel (CH_2Cl_2:MeOH = 20:1) to afford **4** (70 mg, 46%) as white solid. ^{1}H-NMR ($CDCl_3$, 300 MHz) δ 8.76 (d, J = 4.8 Hz, 1H), 7.69 (d, J = 4.8 Hz, 1H), 7.40 (s, 1H), 7.18 (s, 1H), 6.83 (d, J = 8.7 Hz, 1H), 6.65 (s, 1H), 6.58 (d, J = 9.9 Hz, 1H), 6.47 (d, J = 8.6 Hz, 1H), 5.67 (d, J = 9.9 Hz, 1H), 3.98 (s, 3H), 3.91 (s, 3H), 3.86 (s, 3H), 1.41 (s, 6H); HRMS (FAB) calcd for $C_{24}H_{26}NO_5^+$ $[M+H]^+$: 408.1811. Found: 408.1811.

(6,7-Dimethoxyquinolin-4-yl)(5-methoxy-2,2-dimethyl-2H-chromen-6-yl)methyl cyclopropane-carboxylate (5, SL-145)

To a solution of **4** (30 mg, 0.074 mmol) in (4 mL), pyridine (0.02 mL, 0.221 mmol) and cyclopropanecarboxylic acid chloride (0.02 mL, 0.221 mmol) was added at 0 °C and stirred for 30 min. The reaction was terminated by the addition of water and the aqueous phase was extracted with CH_2Cl_2 several times. The combined organic layers were washed with brine, dried over $MgSO_4$, filtered, and evaporated. The residue was purified by flash column chromatography on silica gel

(EtOAc:hexanes = 1:1) to afford **5** (30 mg, 91%) as a pale yellow solid. mp = 80-81 °C; ^{1}H-NMR ($CDCl_3$, 300 MHz) *d* 8.73 (d, 1H, *J* = 4.6 Hz), 7.78 (s, 1H), 7.42 (d, 1H, *J* = 4.8 Hz), 7.39 (s, 1H), 7.34 (s, 1H), 6.98 (d, 1H, *J* = 8.6 Hz), 6.56 (d, 1H, *J* = 10.1 Hz), 6.51 (d, 1H, *J* = 8.4 Hz), 5.66 (d, 1H, *J* = 10.1 Hz), 3.99 (s, 3H), 3.93 (s, 3H), 3.86 (s, 3H), 1.82 (m, 1H), 1.42 (s, 6H), 1.08 (m, 2H), 0.96 (m, 2H); HRMS (FAB) calcd for $C_{28}H_{30}NO_6^+$ $[M+H]^+$ 476.2073. Found: 477.2068; HPLC (Agilent TC-C18, MeOH/H_2O = 90:10, 1 mL/min) t_R = 5.126 min, purity = 97.89%.

2. Purity of SL-145

HPLC conditions:

Column: Agilent TC-C18 (5 μm, 4.6 x 250 mm)

Conditions: Wavelength: 254 nm

Temperature: 20 ^{o}C

Eluent: MeOH: H_2O (90:10)

Flow rate: 1 mL/min

Retention time: 5.126 min

Purity: 97.89%

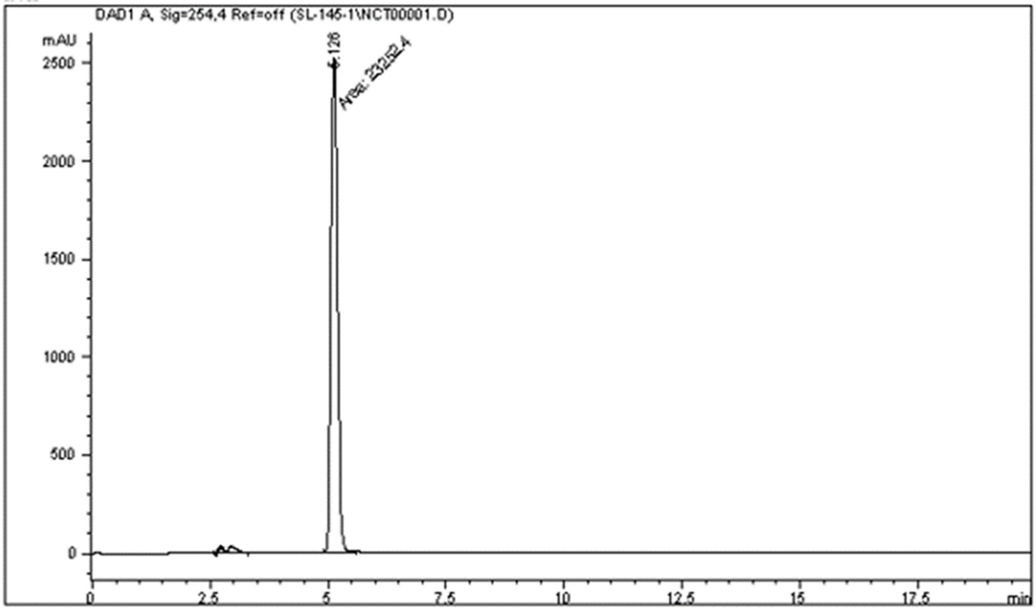

Purity of SL-145 by HPLC Analysis
